# The impact of current treatment modalities on the outcomes of patients with melanoma brain metastases: A systematic review

**DOI:** 10.1002/ijc.32696

**Published:** 2019-11-23

**Authors:** Mark P. van Opijnen, Linda Dirven, Ida E.M. Coremans, Martin J.B. Taphoorn, Ellen H.W. Kapiteijn

**Affiliations:** ^1^ Department of Medical Oncology Leiden University Medical Center Leiden The Netherlands; ^2^ Department of Neurology Leiden University Medical Center Leiden The Netherlands; ^3^ Department of Neurology Haaglanden Medical Center The Hague The Netherlands; ^4^ Department of Radiation Oncology Leiden University Medical Center The Netherlands; ^5^ Leiden University Medical Center, Department of Clinical Oncology Leiden The Netherlands

**Keywords:** brain metastases, melanoma, treatment, outcome

## Abstract

Patients with melanoma brain metastases (MBM) still have a very poor prognosis. Several treatment modalities have been investigated in an attempt to improve the management of MBM. This review aimed to evaluate the impact of current treatments for MBM on patient‐ and tumor‐related outcomes, and to provide treatment recommendations for this patient population. A literature search in the databases PubMed, Embase, Web of Science and Cochrane was conducted up to January 8, 2019. Original articles published since 2010 describing patient‐ and tumor‐related outcomes of adult MBM patients treated with clearly defined systemic therapy were included. Information on basic trial demographics, treatment under investigation and outcomes (overall and progression‐free survival, local and distant control and toxicity) were extracted. We identified 96 eligible articles, comprising 95 studies. A large variety of treatment options for MBM were investigated, either used alone or as combined modality therapy. Combined modality therapy was investigated in 71% of the studies and resulted in increased survival and better distant/local control than monotherapy, especially with targeted therapy or immunotherapy. However, neurotoxic side‐effects also occurred more frequently. Timing appeared to be an important determinant, with the best results when radiotherapy was given before or during systemic therapy. Improved tumor control and prolonged survival can be achieved by combining radiotherapy with immunotherapy or targeted therapy. However, more randomized controlled trials or prospective studies are warranted to generate proper evidence that can be used to change the standard of care for patients with MBM.

**Abbreviations**
ACT: adoptive cell therapy
anti‐PD1: anti‐programmed cell death protein 1
BRAFi: B‐Raf inhibitor
DC: distant control
IPI: ipilimumab
LC: local control
MBM: melanoma brain metastases
MEKi: MEK inhibitor
OS: overall survival
PFS: progression‐free survival
RT: radiotherapy
SRS: stereotactic radiosurgery
SRT: stereotactic radiotherapy
TMZ: temozolomide
WBRT: whole brain radiation therapy

## Introduction

Melanoma is the most aggressive subtype of skin cancer, comprising <5% of all cases. Nevertheless, morbidity is relatively high with approximately 50 000 deaths annually worldwide, especially due to the occurrence of metastases.^1^ After lung and breast cancer, melanoma is the third most common type of cancer likely to metastasize to the brain. An estimated 10–40% of melanoma patients will develop brain metastases.^2^ Prognosis of patients with melanoma brain metastases (MBM) is poor, with an expected overall survival (OS) of only 4 months,^3^, ^4^, ^5^ depending on factors like mutation status.^6^

Conventional therapy for MBM consists of whole brain radiation therapy (WBRT) for multiple metastases and stereotactic radiosurgery (SRS) or radiotherapy (SRT) for limited numbers of metastases. Despite these treatments, outcomes remain poor and the disease burden high. New therapies that could improve patient outcomes are therefore warranted.

The role of conventional chemotherapy and radiation is limited and even comparable to supportive care only in terms of progression‐free survival (PFS).^7^ Since the last decade, several new systemic drugs have been introduced, such as immunotherapy with checkpoint inhibitors like anti‐cytotoxic T‐lymphocyte‐associated protein 4 (ipilimumab [IPI]),^8^, ^9^ anti‐programmed cell death protein 1 (anti‐PD1) (nivolumab and pembrolizumab)^10^, ^11^ or a combination,^12^ and targeted therapy (BRAF, MEK inhibitors [BRAFi, MEKi]).^13^, ^14^, ^15^, ^16^ These therapies can be combined with RT. Currently, the precise impact of available treatment modalities for MBM on tumor‐ and patient‐related outcomes is unknown, as well as the impact of the timing of therapy (i.e., treatment can be given as neoadjuvant, adjuvant or concurrent with other treatment modalities).

This systematic review aimed to describe the impact of current treatment modalities on tumor‐ and patient‐related outcomes of patients with MBM. Given the lack of up to date guidelines on how to treat MBM patients, particularly with the introduction of new therapies, we provide recommendations for the treatment of MBM.

## Methods

### Search strategy

A literature search in the databases PubMed, Embase, Web of Science and Cochrane Library was conducted up to January 8, 2019, using a combination of search terms and synonyms for “melanoma,” brain metastases” and “systemic therapy” (Supplemental S1 for the PubMed search strategy).

All identified abstracts were screened independently by two reviewers (M.P.v.O. and L.D.), and full‐texts of potentially relevant articles were evaluated according to predefined in‐ and exclusion criteria (Supplemental S2). Reference lists of relevant articles were screened for additional eligible articles. Disagreements were resolved in consensus. Preferred Reporting Items for Systematic Reviews and Meta‐Analyses guidelines were followed.^17^

### Data extraction

For each eligible article, information on study design, population characteristics, previously received treatment for MBM, treatment under investigation and outcomes (OS and PFS, local and distant control [LC and DC] and/or toxicity) were extracted. The results are summarized per treatment modality.

### Statistics

Weighted medians or percentages for different outcomes were calculated based on the number of patients included in each study.

## Results

### Search results

**Figure 1:**
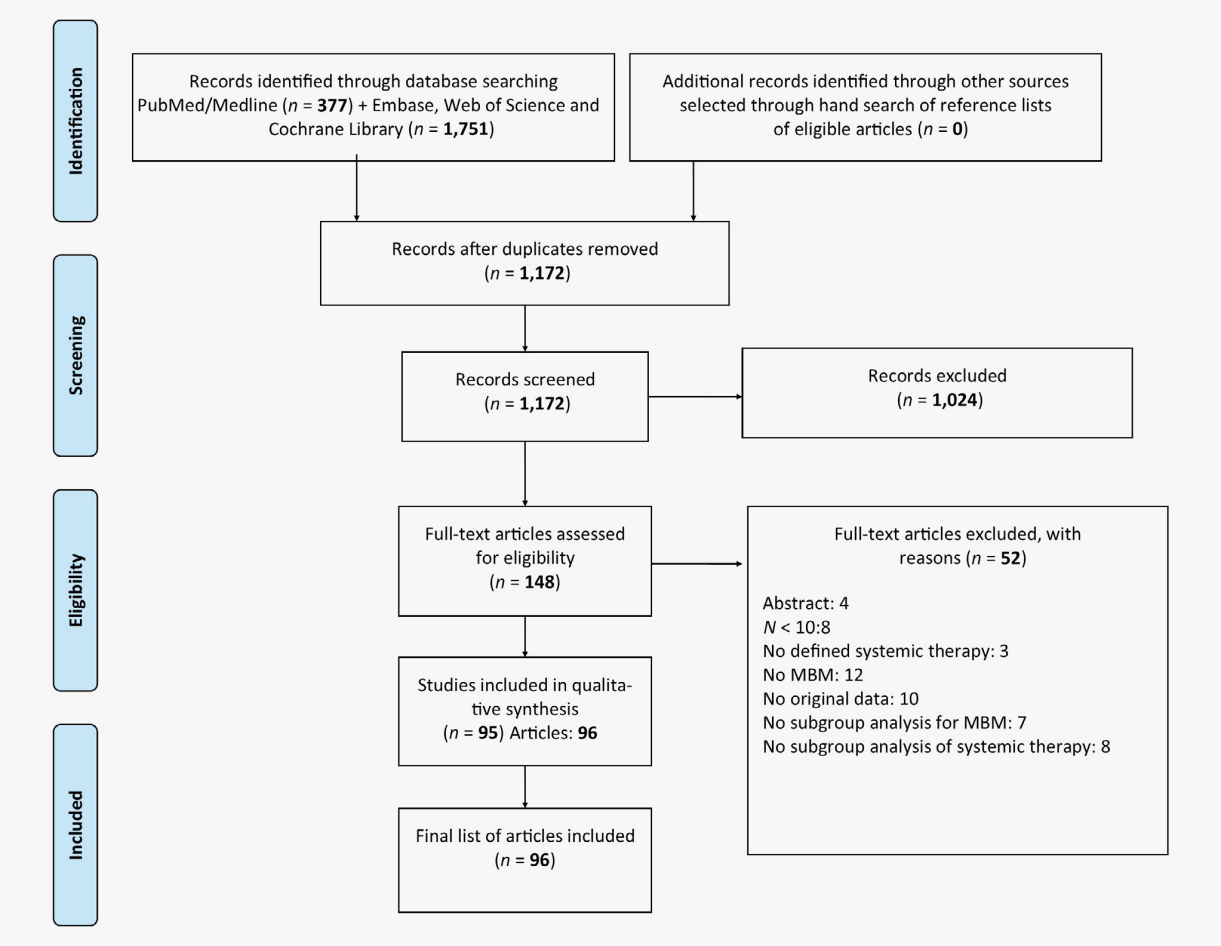
Schematic breakdown of literature search results. Abbreviation: MBM, melanoma brain metastases.

The search strategy resulted in 1,172 unique abstracts. Of these, 148 abstracts were selected for full‐text screening of which 96, comprising 95 studies, were classified eligible according to our predefined criteria. See Figure 1 for an overview of the selection process.

### Study characteristics

Most included studies (79/95, 83%) had a retrospective study design, and the majority (71/95, 75%) was published in 2015 or later. The median number of MBM patients in the studies was 72 (range 10–3,219). Most commonly described treatments were chemotherapy (16/95, 17%), targeted therapy (45/95, 47%) and immunotherapy (60/95, 63%), either or not combined. Of the studies that included targeted therapy or checkpoint inhibitors, vemurafenib (16/45, 36%) and IPI (37/60, 62%) were mostly involved. Radiotherapy (SRS and/or WBRT) as (part of) treatment was investigated in 57/95 (60%) studies. Combined treatment was applied in 63/95 (66%) studies.

The most commonly described outcome was OS. Other outcomes that were reported were control rate (44/95, 46%)—including LC (=no increase in volume of the treated lesions) and DC (=freedom from development of new active disease apart from the treated lesions), PFS (36/95, 38%) and disease‐ and/or drug‐related toxicity (47/95, 49%). See Supplemental S3 for a description of the study characteristics and outcomes of each study.

## Outcomes

### Overall survival

**Figure 2:**
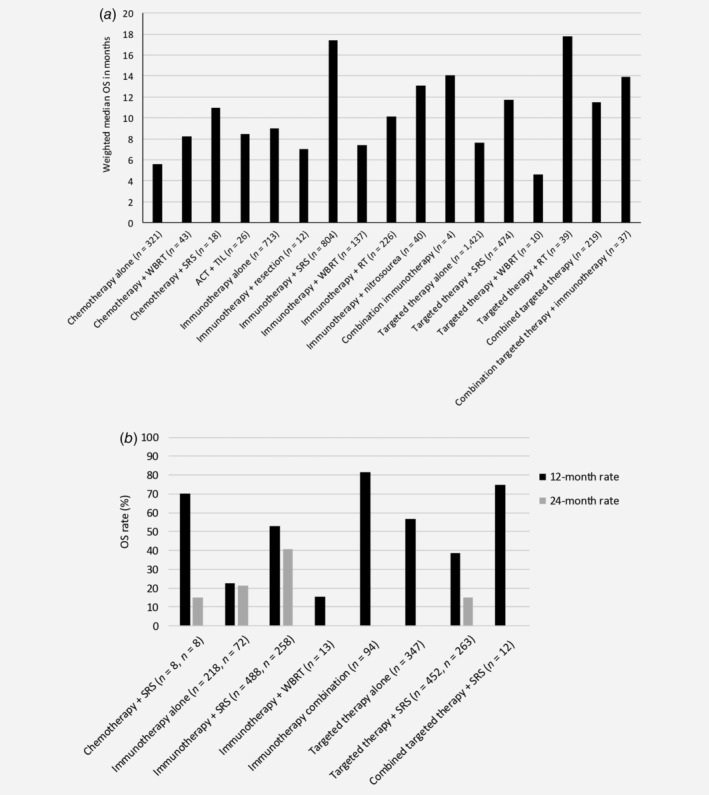
Weighted median overall survival (OS) in months (*a*), and 12‐ and 24‐month OS rates (*b*), separately for the different treatment strategies.

Median OS varies considerably between different treatment modalities, whether given as monotherapy or combined with other modalities, and is significantly shortened in symptomatic patients^18^ and those with higher number of lesions.^19^, ^20^ The OS improved significantly in recent years, particularly with the introduction of targeted and immunotherapy (Figs. 2 *a* and 2 *b*).^21^, ^22^ For some studies, results on OS could not be reported as no subgroup analyses were presented.^18^, ^19^, ^20^, ^23^, ^24^, ^25^, ^26^

The weighted median OS for monotherapy chemotherapy was 5.6 months,^27^, ^28^, ^29^, ^30^, ^31^, ^32^ while the addition of WBRT or SRS resulted in prolonged survival, 8.2^33^, ^34^ and 11^35^ months, respectively, and a 24‐month OS rate of 15%. Similarly, median OS of 8.5 months was found for combined adoptive cell therapy (ACT) with chemotherapy followed by the infusion of autologous tumor‐infiltrating lymphocytes.^36^

Weighted median OS with immunotherapy alone was similar to treatment with chemoradiation, that is, 9.0 months,^30^, ^37^, ^38^, ^39^, ^40^, ^41^, ^42^, ^43^, ^44^, ^45^ with weighted 12‐ and 24‐month OS rates of 22.5%^44^, ^45^ and 21.3%.^45^ The addition of surgery was not effective in terms of OS: median OS of only 7 months.^46^ Combining IPI with nitrosourea, or another immunotherapy (anti‐CLA‐4), did improve median OS to 13.1^47^, ^48^ and 14.1^38^ months, but the combination of immunotherapy with SRS is most effective, with a weighted median OS of 17.4 months.^35^, ^49^, ^50^, ^51^, ^52^, ^53^, ^54^, ^55^, ^56^, ^57^, ^58^, ^59^, ^60^, ^61^, ^62^, ^63^, ^64^, ^65^ Weighted 12‐ and 24‐month survival rates were 52.8%^49^, ^51^, ^52^, ^57^, ^58^, ^63^, ^66^, ^67^, ^68^, ^69^, ^70^, ^71^ and 40.7%^56^, ^57^, ^63^, ^67^, ^68^ with combined immunotherapy and SRS, and 12‐month OS rate was 81.5% for combined IPI and nivolumab.^72^ However, the combination with WBRT resulted in median OS of only 7.4^54^, ^62^, ^73^, ^74^ and with combined SRS/WBRT in a median OS of 10.1^75^, ^76^ months, with a 12‐month OS rate of 15.4%^73^ for combined WBRT+IPI. Timing of RT impacts OS, with higher median OS when RT was given before or during IPI compared to RT given after IPI.^51^, ^56^, ^59^, ^62^, ^75^ Also, the type of immunotherapy given has impact: SRS combined with an anti‐PD1 drug resulted in better survival outcomes than combined with an anti‐CTLA‐4 drug.^38^, ^49^, ^50^, ^58^, ^67^

Survival outcomes for targeted therapy are comparable to those of immunotherapy, with a weighted median OS of 7.6 months,^30^, ^38^, ^77^, ^78^, ^79^, ^80^, ^81^, ^82^, ^83^, ^84^, ^85^, ^86^, ^87^ and a 12‐month OS rate of 56.8%.^83^, ^87^, ^88^ Results on the impact of the presence of specific mutations on the effectiveness of targeted therapy are conflicting.^53^, ^84^ Combining targeted therapy with SRS resulted in similar outcomes as the combination of dabrafenib with trametinib: weighted median OS of 11.7^35^, ^50^, ^89^, ^90^, ^91^, ^92^, ^93^ *versus* 11.5^81^, ^94^, ^95^ months, and 12‐month OS rates of 38.7%^67^, ^89^, ^91^, ^92^, ^93^, ^96^, ^97^ *versus* 48.4%,^94^ respectively. The 24‐month OS rate for targeted therapy with SRS was 15.2%.^91^, ^93^ Again, median OS was worse in patients also treated with WBRT, 4.6 months.^98^ With respect to timing, SRS before BRAFi resulted in significantly prolonged survival compared to SRS after BRAFi or concurrently to SRS.^91^ Lastly, combining targeted therapy (BRAFi/MEKi) with immunotherapy resulted in a weighted median OS of 13.9 months,^30^, ^38^ and the combination with SRS in a 12‐month OS rate of 75%.^67^

### Progression‐free survival

**Figure 3:**
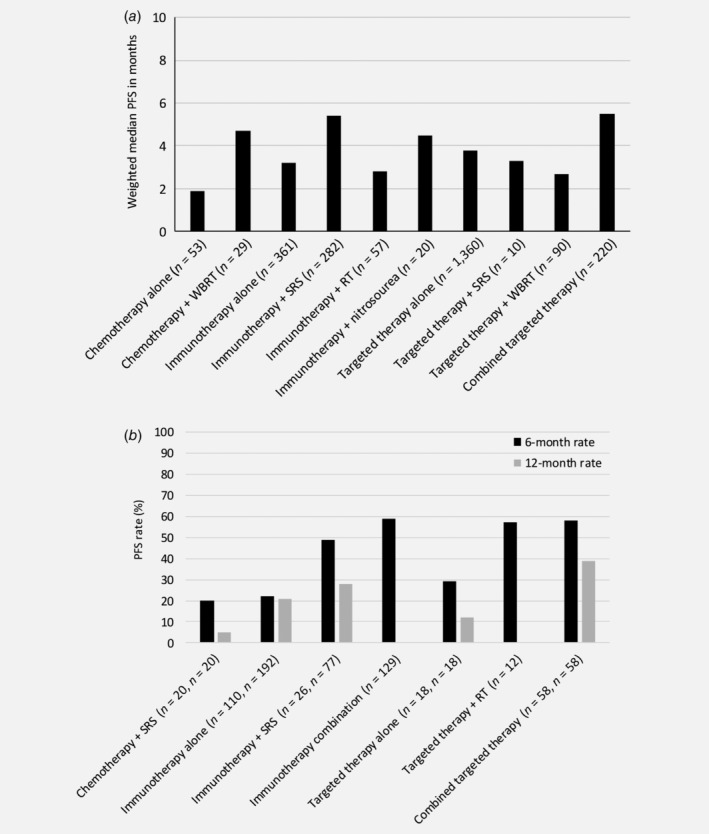
Weighted median progression‐free survival (PFS) in months (*a*), and 6‐ and 12‐month PFS rates (*b*), separately for the different treatment strategies.

Treatment with temozolomide (TMZ) chemotherapy alone resulted in PFS of 1.9 months,^28^ which improved to 4.7 months if combined with WBRT.^33^ The 6‐ and 12‐month PFS rates were 20% and 5% for combined chemotherapy with SRS, respectively (Fig. 3).^67^

Treatment with immunotherapy monotherapy resulted in weighted median PFS of 3.1 months,^39^, ^41^, ^43^, ^44^, ^45^, ^99^ and weighted 6‐month and 12‐month PFS rates of 22%^41^, ^44^, ^67^ and 21%,^44^, ^67^ respectively. Patients who received previous treatment for their MBM had better median PFS (5.0 *vs*. 1.2 months) compared to previously untreated patients.^99^ Combined treatment slightly improved PFS results. Immunotherapy with SRS or RT resulted in a weighted median PFS of 5.4^55^, ^57^, ^58^, ^59^, ^61^, ^100^ and 2.8^75^ months, respectively. The median immune‐related PFS for combined immunotherapy with nitrosourea (i.e., IPI + fotemustine) was 4.5 months.^47^, ^74^ Results regarding the optimal timing of combined immunotherapy and RT differed between studies, but the weighted median PFS was 9.2 months if SRS was given before or during immunotherapy *versus* 4.2 months when SRS was given nonconcurrently.^43^, ^57^, ^59^, ^75^ Finally, combining immunotherapies (i.e., IPI and nivolumab) resulted in high 6‐month intra‐ and extracranial PFS rates (64.2% and 75.9%, respectively).^72^

The weighted median PFS for patients treated with targeted monotherapy was 3.8 months,^77^, ^78^, ^79^, ^80^, ^81^, ^82^, ^83^, ^84^, ^85^, ^87^, ^101^ which is similar to treatment with immunotherapy alone, but could be increased to 5.5 months by combining BRAFi+MEKi.^81^, ^94^, ^95^ Patients with a specific BRAF mutation (Val600Lys) who received previous local treatment and were treated with dabrafenib had a similar median PFS as chemotherapy alone (1.9 months^84^), but was higher with a Val600Glu mutation and treatment with dabrafenib plus trametinib (7.2 months^94^). Initiating targeted therapy (mitogen‐activated protein kinase inhibitor) after the occurrence of MBM is more effective in preventing progression of metastases when compared to targeted treatment that was already initiated prior to the occurrence of MBM, 7.1 *versus* 2.1 months, respectively.^87^ Previous treatment for MBM did not change PFS in patients treated with targeted therapy.^85^ Combination of targeted therapy with WBRT or SRT resulted in weighted median PFS of 3.3^98^ and 2.7^92^ months, respectively, or 6‐month freedom‐from‐new‐MBM rate of 57%.^102^ The 6‐ and 12‐month PFS rates for patients treated with SRS plus targeted therapy (BRAFi) was 29% and 12%, respectively, which increased to 58% and 39% when BRAFi was combined with another targeted therapy (i.e., MEKi) as addition to SRS.^67^ In BRAF‐mutated patients, combined SRS + BRAFi resulted in a median PFS of 3.9 *versus* 1.7 months in those without a mutation (*p* = 0.02).^92^

### Control rate

**Figure 4:**
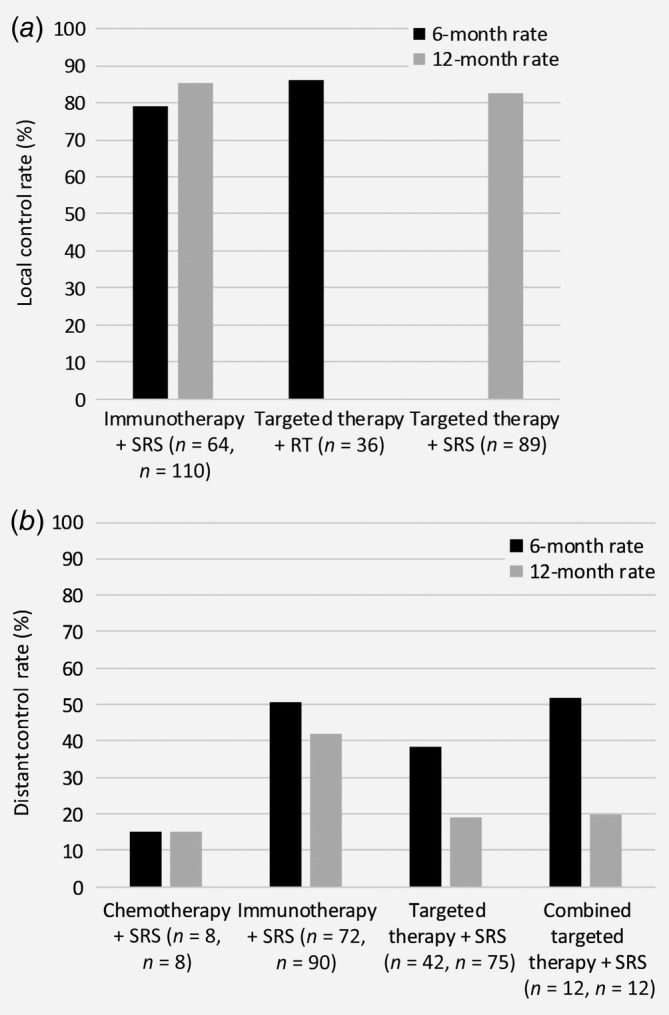
Weighted local (*a*) and distant (*b*) control rates, separately for the different treatment strategies.

Mean 6‐ and 12‐month LC rates were similar when RT combined with either immunotherapy (79%^49^, ^70^, ^103^ and 85.4%,^49^, ^66^, ^69^, ^71^ respectively) or targeted therapy (86.3%^89^, ^102^ and 82.4%,^66^, ^89^, ^93^, ^96^, ^97^ respectively) (Fig. 4 *a*). MBM response rate was 22% with pembrolizumab only.^104^ However, when immunotherapy (pembrolizumab or IPI) was combined with SRS, LC rates were higher, ranging between 68% and 94.8%.^55^, ^58^, ^63^, ^105^ Of note, tumor control with combined therapy was lower in hemorrhagic *versus* nonhemorrhagic metastases (43% *v*. 83%, respectively^68^). Although not significant, local failure was lower when SRS was given concurrently with IPI *versus* noncurrent administration, 10% *versus* 19%.^52^ The overall response rate for vemurafenib monotherapy, defined as combined intra‐ and extracranial response, was reached in 10/24 (42%) patients in an open‐label Phase I trial.^79^ Targeted therapy with or without SRS resulted in a control rate of 92.5% in another study.^92^ Mean intracranial control rate was 37.8% and 55.8% for treatment with immunotherapy^40^, ^41^, ^43^ or targeted therapy^81^, ^88^, ^106^ alone, which increased to 52.5% when nivolumab and IPI were combined^41^, ^72^ and to 56.8% when dabrafenib and trametinib were combined.^81^, ^94^ Combined SRS with immunotherapy resulted in intracranial disease response after a median of 5.4 months,^51^ and was higher when IPI was administered before RT instead of after (40% *v*. 16.7%), although this was not statistically significant.^62^ If SRS is given after IPI, significantly prolonged intracranial control rates are reached when this is done within 5.5 months: 8.4 *versus* 3.6 months.^107^

Only one small study evaluated the DC rates (Fig. 4 *b*) in MBM patients for chemotherapy combined with SRS, showing 6‐ and 12‐month rates of 15% and 5%, respectively.^67^ The overall DC rate was 34.2% with immunotherapy alone,^42^ and intracranial response rates ranged between 18.8% and 24%.^41^ DC rates increased when immunotherapy was combined with RT, with mean 6‐ and 12‐month DC rates of 50.7%^49^, ^67^ and 42%,^49^, ^66^, ^67^ respectively. One small study even found a 24‐month DC rate of 95.4% after combined treatment of SRS with immunotherapy.^57^ Similarly, intracranial response rate was 48.6% when IPI and nivolumab were combined.^41^ DC rates were lower for the combination of targeted therapy with RT, that is, mean 6‐ and 12‐month DC rates of 38.6%^67^, ^89^ and 18.9%.^66^, ^67^, ^89^, ^96^ Nevertheless, combining BRAFi + MEKi and SRS resulted in higher 6‐ and 12‐month DC rates, 52% and 20%, respectively,^67^ or intracranial response rates between 44% and 65% after a median of 8.5 months follow‐up.^94^ The 12‐month distant failure rate could be significantly reduced for BRAF‐mutated patients treated with BRAFi, from 95% to 68%.^26^ Control rate can also be expressed in the number of new MBM while under treatment. One study found a significantly higher percentage of patients with new MBM under BRAFi treatment compared to the control group without BRAFi (60% *vs*. 15%, respectively); however, mean size of new lesions was smaller.^108^ The occurrence of new MBM appears to depend on the timing of treatment with vemurafenib: BRAF‐mutated patients without MBM who were treated with vemurafenib had a significantly decreased chance of developing MBM (incidence ratio = 0.51) when compared to patients who did not receive upfront vemurafenib.^109^

### Toxicity

Treatment with chemotherapy obviously resulted in significantly more toxicity when compared to best supportive care alone,^31^ with moderate to severe toxicity in up to 30% of patients.^28^, ^31^ Grade 4 toxicities were uncommon (<2%).^28^ Grade 3 toxicity ranged from 3.4% for fatigue, neutropenia and lymphedema to 13.8% for thrombocytopenia when TMZ was combined with WBRT.^33^ Hemorrhage occurred in 5.9% of patients treated with combined chemotherapy and ACT.^36^

Mild to moderate toxicity was common with immunotherapy alone,^45^, ^110^ but Grade 3/4 toxicity was relatively low (weighted mean percentage of 8.4%).^41^, ^44^, ^104^ Although two studies showed that the combination of immunotherapy, IPI^55^, ^111^ or anti‐PD1,^55^ with RT resulted in significantly more brain toxicity than RT alone, the overall risk of Grade 3/4 toxicities was similar to that of immunotherapy alone, i.e. weighted mean of 8.1%.^51^, ^64^, ^68^, ^69^, ^73^, ^74^, ^75^, ^112^, ^113^ However, combining immunotherapy with nitrosourea resulted in a treatment‐related Grade 3/4 toxicity of 55%.^47^ Similarly, combining immunotherapies resulted in significant Grade 3/4 toxicity: weighted mean of 54.7%.^41^, ^72^ Radiation necrosis was observed in 30.4% of patients treated with pembrolizumab in one small study^39^ and in an average of 13.8% of patients receiving immunotherapy combined with RT,^55^, ^58^, ^59^, ^61^, ^71^, ^100^ and 11.6% of lesions.^63^, ^65^ Similarly, intracranial hemorrhage was observed in 23.3% of patients receiving combined treatment^55^, ^70^, ^71^ and intratumoral hemorrhage in all patients in one small study.^73^

Patients treated with targeted therapy alone experience Grade 3/4 toxicity more often than with immunotherapy alone, that is, an average of 37.2% of patients,^78^, ^82^, ^84^, ^85^ with skin lesions/rash being most common.^83^ The combination of targeted therapies (dabrafenib and trametinib) resulted in increased Grade 3/4 toxicity (48%).^94^ Combining vandetanib with WBRT resulted in Grade 3/4 toxicity rate of 50%, which was similar to WBRT with placebo.^98^ The average hemorrhage rate was similar when treated with targeted therapy alone or when combined with RT (11.2%^84^, ^96^ *vs*. 12.7%^91^, ^92^, ^93^, ^97^). Radiation necrosis occurred in an average of 16% of patients.^97^, ^102^

## Discussion

In an attempt to improve the survival of MBM patients, several new systemic therapies have been introduced in the last decade, including targeted therapy and immunotherapy. This review showed that these new treatment modalities have been administered as monotherapy, but also in combination with conventional treatment modalities such as chemotherapy and RT. Not only the type of treatment was found to have an impact on treatment outcomes, also the timing of drug administration appears important. It should be noted that survival is not only determined by the presence and treatment of MBM, but also by the status of extracranial disease. A major limitation is that most studies included in this review are retrospective studies and that studies vary considerably in terms of the included patient population (e.g., performance status and extent of intra‐ and extracranial disease) and type of treatment used for these patients (e.g., previously SRT was only used for patients with a limited number of MBM and nowadays SRT is also used for patients with >10 MBM with limited total metastatic volume), hampering the conclusions that can be drawn. Nevertheless, we will provide recommendations for the treatment of MBM patients based on the available literature. These recommendations can be updated if better studies are published. It is important that the impact of treatment on all outcomes is considered. Although tumor‐related outcomes such as LC rate may be important to evaluate treatment effectiveness, this outcome may be less important for a patient. For example, the tumor may respond well, but if a patient is experiencing considerable treatment toxicity, this treatment may be less meaningful for that patient.

Treatment with immuno‐ and targeted therapy are preferred over treatment with chemotherapy alone as they improve both OS and PFS, particularly in combination with RT. A drawback is that significantly more radiation necrosis and Grade 3/4 toxicities were observed for combined treatment. However, it is difficult to discriminate between disease‐ and drug‐related toxicities. It is important to consider the timing of administrating the different treatment modalities. RT delivered before or during immune‐ or targeted therapy seems to result in longest OS^51^, ^56^, ^59^, ^62^, ^75^, ^91^ and PFS.^43^, ^57^, ^59^, ^75^

Although the results of the included studies are variable and, in some cases, contradictory, the general consensus seems to be that combined treatment of RT with immune or targeted therapy resulted in the highest control rates. Particularly the combination with BRAFi and MEKi seems valuable, which was also correlated with higher OS and PFS.^67^ Despite the fact that the safety of combining RT and targeted therapy has been established in several studies,^92^, ^114^ concerns have been raised with respect to possible increased toxicity, particularly with BRAF‐inhibitors.^115^ Further studies addressing the toxicity of this combined treatment are therefore warranted, including assessment of the impact on radiation necrosis, cognition and HRQoL.^116^ The combination of RT and immunotherapy, however, has not only proven to be efficacious, but also safe in terms of neurotoxicity.^61^, ^103^, ^113^, ^117^, ^118^, ^119^ Another issue is that targeted treatment with BRAFi and MEKi may result in resistance after long‐term use, suggesting that the best long‐term responses can be achieved by using immunotherapy after rapid tumor reduction with BRAFi/MEKi. The highest level of evidence for treatment of asymptomatic and untreated MBM patients with immunotherapy is provided by Long *et al*. and Tawbi *et al*., two relatively large (randomized) Phase II trials, showing that the combination of IPI and nivolumab resulted in relatively high intracranial control rates.^41^, ^72^ Recently, a randomized Phase II trial comparing IPI and nivolumab with concurrent intracranial SRT *versus* IPI and nivolumab alone in patients with asymptomatic, untreated MBM has opened for recruitment.^120^ Results of this trial will contribute to further improvement of treatment recommendations for this patient population.

To conclude, MBM patients seem to benefit most from treatment with targeted and immunotherapy, preferably combined with RT to create a synergistic effect, although toxicity may be increased with this strategy. Nevertheless, based on the available data, it is difficult to recommend one specific treatment for MBM patients. The exact treatment should therefore be based on the characteristics of individual patients (e.g., genetic profile and other tumor‐ and patient‐related characteristics), as well as their treatment preference. In order to achieve further improvements in the treatment of patients with MBM, it is essential to study the novel immunotherapies and targeted therapies, whether or not combined with RT (particularly SRS), in more (randomized controlled) trials to create more evidence‐based guidance. Finally, future research may emerge new targets for treatment which can also contribute to more patient‐specific treatments that can subsequently improve outcomes.

## Supporting information


**Appendix S1:** Supporting information

**Appendix S2:** Supporting information

**Appendix S3:** Supporting information
