# Supplementary material for: The impact of current treatment modalities on the outcomes of patients with melanoma brain metastases: A systematic review
Source: Int J Cancer. 2019 Nov 23;146(6):1479–89. doi: 10.1002/ijc.32696 (PMC7004107; doi:10.1002/ijc.32696)
Supplement: Supplementary file 1 — Appendix S1: Supporting information [file IJC-146-1479-s001.doc]

**Search strategy PubMed**

**("Brain Neoplasms/secondary"[majr] OR (("Brain Neoplasms"[majr] OR "brain"[ti] OR "brains"[ti] OR "intracranial"[ti] OR "cerebral"[ti]) AND ("Neoplasm Metastasis"[majr] OR "metastases"[ti] OR "metastasis"[ti] OR "metastatic"[ti] OR metasta*[ti]))) AND ("Melanoma"[mesh] OR "melanoma"[tw] OR "melanomas"[tw] OR melanoma*[tw]) AND ("systemic therapy"[tw] OR "systemic therapies"[tw] OR systemic chemotherap*[tw] OR systemic immunotherap*[tw] OR systemic anti*[tw] OR systemic administr*[tw] OR systemic drug*[tw] OR systemic gene*[tw] OR systemic treat*[tw] OR "Combined Modality Therapy"[Mesh:NoExp] OR combined modalit*[tw] OR "Neoadjuvant Therapy"[Mesh] OR "Neoadjuvant Therapy"[tw] OR "Neoadjuvant Therapies"[tw] OR multimodal treat*[tw] OR multimodal therap*[tw] OR "Antineoplastic Agents" [Pharmacological Action] OR "vemurafenib"[Supplementary Concept] OR "vemurafenib"[tw] OR "Zelboraf"[tw] OR "ipilimumab"[Supplementary Concept] OR "ipilimumab"[tw] OR "Yervoy"[tw] OR "temozolomide"[Supplementary Concept] OR "temozolomide"[tw] OR "Temodal"[tw] OR "Temodar"[tw] OR "nivolumab"[Supplementary Concept] OR "nivolumab"[tw] OR "Opdivo"[tw] OR "pembrolizumab"[Supplementary Concept] OR "pembrolizumab"[tw] OR "lambrolizumab"[tw] OR "Keytruda"[tw] OR chemotherap*[tw] OR "Drug Therapy"[mesh] OR "drug therapy"[subheading] OR "drug"[tw] OR "drugs"[tw] OR "Immunotherapy"[mesh] OR immunotherap*[tw] OR "targeted therapy"[tw] OR "targeted treatment"[tw] OR targeted therap*[tw] OR targeted treat*[tw] OR "checkpoint inhibitor"[tw] OR "checkpoint inhibitors"[tw] OR checkpoint inhibit*[tw] OR "pd1"[tw] OR** "Programmed Cell Death 1"[tw] OR **"pd-1"[tw] OR "antiPD1"[tw] OR "antiPD-1"[tw] OR** "Programmed Cell Death 1 Receptor"[Mesh] OR "Antigens, CD274"[Mesh] OR "CD274"[tw] OR **"braf inhibitor"[tw] OR "braf inhibitors"[tw] OR braf inhibit*[tw] OR "encorafenib" [Supplementary Concept] OR "encorafenib"[tw] OR "BFD-22" [Supplementary Concept] OR "BFD-22"[tw] OR "BGB-283" [Supplementary Concept] OR "BGB-283" [tw] OR "Antibodies, Monoclonal"[Mesh] OR "Monoclonal Antibody"[tw] OR "Monoclonal Antibodies"[tw] OR monoclonal antibod*[tw] OR "Adalimumab"[tw] OR "Bevacizumab"[tw] OR "Certolizumab"[tw] OR "Pegol"[tw] OR "Cetuximab"[tw] OR "Denosumab"[tw] OR "Natalizumab"[tw] OR "Omalizumab"[tw] OR "Palivizumab"[tw] OR "Ranibizumab"[tw] OR "Trastuzumab"[tw] OR "Ustekinumab"[tw] OR "Muromonab-CD3"[tw] OR "Rituximab"[tw] OR "Infliximab"[tw] OR "Antibody"[tw] OR "antibodies"[tw] OR "mAb"[tiab] OR "mAbs"[tiab] OR "dabrafenib"[tw] OR "dabrafenib" [Supplementary Concept] OR "MEK inhibitor I" [Supplementary Concept] OR (("MEK"[tiab] OR MEK*[tiab] OR MAPK*[tiab] OR MAP kinase*[tw]) AND ("inhibitor"[tw] OR "inhibitors"[tw] OR "inhibition"[tw])) OR "RO5126766" [Supplementary Concept] OR "RO5126766" [tw] OR "Cobimetinib"[tw] OR "GDC-0973" [Supplementary Concept] OR "Binimetinib"[tw] OR "MEK162" [Supplementary Concept] OR "trametinib" [Supplementary Concept] OR "trametinib" [tw]) NOT (("Animals"[mesh] OR "mice"[ti] OR "mouse"[ti] OR "rats"[ti] OR "rat"[ti]) NOT "Humans"[mesh]) NOT (("case reports"[ptyp] OR "case report"[ti]) NOT "Clinical Study"[ptyp])**
