# Supplementary material for: The impact of current treatment modalities on the outcomes of patients with melanoma brain metastases: A systematic review
Source: Int J Cancer. 2019 Nov 23;146(6):1479–89. doi: 10.1002/ijc.32696 (PMC7004107; doi:10.1002/ijc.32696)
Supplement: Supplementary file 2 — Appendix S2: Supporting information [file IJC-146-1479-s002.doc]

*Inclusion and exclusion criteria*

Original articles published since 2010 in English, German or Dutch in peer-reviewed journals, and describing tumor- and patient-related outcomes of adult MBM patients treated with clearly defined systemic therapy, were included. Case reports and case series (<10 MBM patients), reviews, animal studies, studies with children, or studies not presenting the results for MBM patients separately were excluded. There were no restrictions with respect to the study design.
