# Supplementary material for: The impact of current treatment modalities on the outcomes of patients with melanoma brain metastases: A systematic review
Source: Int J Cancer. 2019 Nov 23;146(6):1479–89. doi: 10.1002/ijc.32696 (PMC7004107; doi:10.1002/ijc.32696)
Supplement: Supplementary file 3 — Appendix S3: Supporting information [file IJC-146-1479-s003.doc]

|  | **Article** | **Study Design** | **Population** | **Previously treated BM (*N*, (%))** | **Treatment (*N*, %)** | **Overall Survival** | **Local Control** | **Distant Control** | **Progression Free Survival** | **Toxicity** |
| --- | --- | --- | --- | --- | --- | --- | --- | --- | --- | --- |
| 1 | Acharya et al., 2017 | Retrospective review of database | 72 MBM-patients  *SRS 38/72*  - Median (range) age: 56 (31-81)  - Male/female (*N*): 31/7  *SRS + IT 18/72*  - Median (range) age: 61 (36-86)  - Male/female (*N*): 12/6  *SRS + BRAF/MEK 16/72*  - Median (range) age: 52 (31-65)  - Male/female (*N*): 6/10 | Steroid use prior to SRS:  SRS: 22/121 MBMs (18)  SRS + IT: 7/48 MBMs (15)  SRS + BRAF/MEK: 17/64 MBMs (15) | SRS  SRS combined with anti-PD1 (6%), anti-CTLA-4 (65%) or both (29%)  SRS combined with BRAFi, MEKi or both (44%) | *SRS:*  1-year OS: 36%  *SRS + IT:*  1-year OS: 58%, *P*=0.016 | 1-year LC rates:  *SRS:*  66%  *SRS + IT:*  85%  *SRS + BRAF/MEK:*  72%  *P*=0.044 | 1-year DC rates:  *SRS:*  11.5%  *SRS + IT:*  60%  *SRS + BRAF/MEK:*  10%  *P* <0.001 |  | *SRS:*  - Seizures: 1/38 (2.6%)  *SRS: BRAFi:*  - Seizures: 1/16 (6.3%) |
| 2 | Ahmed et al., 2016 | Retrospective review of database | 96 MBM-patients  - Median (range) age: 61.8 (18-90)  - Male/female (*N*): 60/36 | Systemic: 39/96 (40.6) | SRS combined with:  - Anti-PD-1 (Nivolumab (14, 67) or Pembrolizumab (7, 33))  - Anti-CTLA-4 (IPI (25, 100))  - BRAF/MEKi (Dabrafenib and Trametinib (12, 100))  - BRAFi (Vemurafenib (18, 100))  - Chemotherapy (Carboplatin and Paclitaxel (8, 40)) | Median (range) OS*: 8.9 months (0.47-48)  6- and 12-month OS from the date of cranial metastases:  - Anti-PD-1: 81%/66%  - Anti-CTLA-4: 84%/50%  - BRAF/MEKi: 83%/75%  - BRAFi: 71%/29%  - Chemotherapy: 70%/15% | 6- and 12-month LC rates:  89%/83%  (*P*=0.25) | 6- and 12-month DC rates from the date of SRS:  Overall: 36%/18%  - Anti-PD-1: 61%/38%  - Anti-CTLA-4: 26%/21%  - BRAF/MEKi: 52%/20%  - BRAFi: 30%/8%  - Chemotherapy: 15%/5% | Median (range)*: 3.4 (0.47-45.9) months  6- and 12-month systemic PFS from the date of SRS:  - Anti-PD-1: 41%/41%  - Anti-CTLA-4: 36%/27%  - BRAF/MEKi: 58%/39%  - BRAFi: 29%/12%  - Chemotherapy: 20%/5% |  |
| 3 | Ahmed et al., 2015 | Retrospective review of database | 24 MBM-patients  - Median (range) age: 58.5 (21-88)  - Male/female (*N*): 19/5 | CT: 7/24 (29.2)  IL-2: 1/24 (4.2)  IPI: 8/24 (33.3)  MEKi: 6/24 (25.0)  Anti-PD-1: 2/24 (8.3) | SRS combined with Vemurafenib | Patients died during FU: 15/24 (62.5%)  Median (range) OS*: 7.2 (1.5-26.8) months  6- and 12-month OS:  - DS-GPA class 1-1.5: 61%/38%  - DS-GPA class 2: 100%/83% | 6- and 12-month LC rates: 92%/75%  Median (range) time to local failure: 6.1 (2-20.1) months* | 6- and 12-month DC rates: 45%/23%  Distant brain failure (n, %): 14/24, 58.3  Median (range) time to distant brain failure: 3.4 (1.9-16.1) months* |  |  |
| 4 | Ahmed et al., 2016 | Retrospective review from a phase 1 study | 26 MBM-patients  - Median (range) age: 54.5 (33-79)  - Male/female (*N*): 17/9 | CT: 9/26 (34.6)  IL-2: 4/26 (15.4)  IPI: 13/26 (50.0)  BRAF/MEKi: 2/26 (7.7)  BRAFi: 2/26 (7.7) | SRS combined with Nivolumab | Patients died during FU: 16/26 (62%)  Median (range) OS*: 11.8 (0.5-33.9) months  6- and 12-month OS*: 78%/55% | 6- and 12-month LC rates: 91%/85%  6- and 12-month LC lesions undergoing SRS (excluding FSRT): 89%/82%  Median (range) time to failure: 6 (4.5-8.0) months | 6- and 12-month DC rates: 66%/53%  Median (range) time to distant failure: 4.6 (2.6-29.1) months |  |  |
| 5 | Amaral et al., 2019 | Retrospective review of database | 163 MBM-patients  - Median (range) age: 63 (54.0-74.0)  - Male/female (*N*): 93/70 | Yes: 81/163 (49.7) | SRS alone and combined with IT (64/163), TT (44/163) or CT (18/163) | *Prior systemic therapy:*  - Yes: median OS = 6 months  - No: median OS = 10 months  *P*=0.005  Median OS:  - IT: 13 months  - TT: 7 months  - CT: 7 months  - No Tx: 3 months  *P*<0.0001  Median OS:  - SRS + IT: 25 months  - SRS + TT: 14 months  - SRS + CT: 11 months  *P*<0.0001  1-year OS:  - IT: 53.1%  - TT: 29.5%  - CT: 11.1%  - No Tx: 10.8%  2-year OS:  - IT: 37.5%  - TT: 8.4% |  |  |  |  |
| 6 | An et al., 2017 | Retrospective review of database | 99 MBM-patients  - Median (range) age: 62.5 (16.4-89.7)  - Male/female (*N*): 69/30 | No prior radiation and IT  Additional therapy:  - anti-PD1: 23/99 (23.2)  - BRAFi: 9/99 (9.1)  - CT: 38/99 (38.4) | SRS + IPI | Median OS:  - SRS > 5.5 months after IPI: SRS < 5.5 months after IPI = HR 1.78  *P*=0.129 | Median intracranial control rates:  - SRS < 5.5 months after IPI (*N*=51): 8.43 months  - SRS > 5.5 months after IPI (*N*=20): 3.63 months  *P*=0.02  1-year intracranial rates:  - SRS < 5.5 months after IPI: 51%  - SRS > 5.5 months after IPI: 25% |  |  |  |
| 7 | Anderson et al., 2017 | Retrospective review of database | 21 MBM-patients:  - Median (range) age: 67 (32-84)  - Male/female (*N*): 14/7 | Systemic therapy: 13/21 (61.9)  IPI: 10/13 (76.9) | SRS + pembrolizumab |  | Median intracranial response rates:  - SRS + Pembro: 70% (16/23 lesions)  - SRS + IPI: 32% (10/31 lesions)  - SRS: 22% (5/27 lesions) |  |  | - Grade 3 edema resulting in confusion: 1/21 (4.8%) |
| 8 | Azer et al., 2014 | Retrospective review of database | 23 MBM-patients  - Median (range) age: 58 (23-82)  - Male/female (*N*): 15/8 | Surgery: 3/23 (13.0)  SRS: 1/23 (4.3)  WBRT: 2/23 (8.7)  Combination: 5/23 (21.7) | Dabrafenib | Patients died during FU: 17/23 (73.9%)  Median (95% CI, range) OS: 36.6 (22.2-50.9, 14.4-103.1) weeks |  |  | Median (95% CI, range) overall PFS: 16.3 (14.1-18.5, 4.0-44.9) weeks |  |
| 9 | Bauer-Nilsen et al., 2018 | Retrospective review of database | 134 MBM-patients  - Median (range) age: 61.7 (21.2-84.9)  - Male/female (*N*): 92/42 | *Solid:*  WBRT: 19.0%  CT: 63.0%  *Hemorrhagic:*  WBRT: 23.9%  CT: 72% | SRS combined with IT (15% for solid, 31% for hemorrhagic) | Median OS:  - 1-year: 42%  - 2-year: 31%  - 3-year: 12% | 6-months LC:  - hemorrhagic metastases: 43%  - solid metastases: 83%  *P*<0.001  Time to local tumor progression:  - Prior WBRT: HR = 1.62, *P*=0.003  - Prior CT: HR = 0.69, *P*=0.011 |  |  | No significant difference between the groups |
| 10 | Blank et al., 2017 | Open-label study | 3219 patients, 753 MBM-patients  - Median (range) age: 55 (13-95)  - Male/female (*N*): 1822/1397 | RT: 1137/3219 (35)  Surgery: 3159/3219 (98)  CT: 1406/3219 (44)  IPI: 286/3219 (9)  BRAFi: 19/3219 (1)  MEKi: 71/3219 (2) | Vemurafenib | Median (95% CI) OS in MBM: 7.4 (6.9-8.0) months |  |  | Median (95% CI) PFS in MBM: 3.7 (3.7-3.9) months | Grade 3/4 AE:  - Any: 1176/3219 (37%)  - Arthralgia: 102/3219 (3%)  - Keratoacanthoma: 253/3219 (8%)  - SCC of the skin: 256/3219 (8) |
| 11 | Bordia et al., 2017 | Retrospective review of database | 98 MBM-patients:  - Median (range) age: unknown  - Male/female (*N*): unknown | No prior treatment | - Surgery: 24%  - SRS: 49%  - WBRT: 25%  - BRAFI: 17%  - IPI: 26%  - CT: 32% | Median OS:  - Single lesion (*N*=29): 9.1 months  - >1 lesion (*N*=69): 4.9 months  *P*=0.002  BRAFi prognostic factor, *P*=0.002 |  |  |  |  |
| 12 | Choong et al., 2017 | Retrospective review of database | 108 MBM-patients  - Median (range) age: 64.3 (17.3-87.3)  - Male/female (*N*): 75/33 | WBRT (unknown *N* and %) | SRS combined with:  - Anti-PD-1 (11, 10.2)  - Anti-CTLA-4 (28, 26.0)  - BRAF/MEKi (39, 36.1)  - MEKi (1, 1.0)  - No (26, 24.1) | Patients died during FU: 58/108 (53.7%)  Median (95% CI) OS: 14.2 (8.8-20.4) months  6- and 12-month (95% CI) OS: 56% (45-65)/34% (23-46)  Median (95% CI) OS – specified:  - Anti-PD-1: 20.4 (8.8-N/A) months  - Anti-CTLA-4: 7.5 (4.4.15.6) months  - BRAFi ± MEKi: 17.8 (11.8-N/A) months  Median OS without systemic therapy: 10.8 months | 6- and 12-month brain control (BC): 67%/48%  Median (95% CI) duration of BC:  - Anti-PD-1: 12.7 (5.5-N/A) months  - Anti-CTLA-4: 7.5 (4.0-15.6) months  - BRAF/MEKi: 12.7 (8.3-18.5) months |  |  |  |
| 13 | Cohen-Inbar et al., 2017 | Retrospective review of database | 46 MBM-patients, 32/46 group A, 14/46 group B  Group A (SRS before or during IPI):  - Median (range) age: 62.0 (24.3-83.6)  - Male/female (*N*): 23/9  Group B (SRS after IPI):  - Median (range) age: 66.7 (48.4-83.6)  - Male/female (*N*): 10/4 | Group A:  - Systemic: 15 (46.9)  - WBRT: 3 (9.4)  Group B:  - Systemic: 11 (78.6)  - WBRT: 1 (7.1) | SRS combined with IPI | Median (range) OS after SRS, entire cohort: 13.4 (1.2-43.2) months  *Group A*  Median (range) OS: 13.8 (2.7-43.2) months  6- and 12-month OS: 81.3%/59.2%  *Group B*  Median (range) OS: 6.4 (1.2-24.5) months  6- and 12-month OS: 57.1%/33.3% | Intracranial disease response (range):  - Overall: 5.4 (0.4-34.7) months  - Group A: 7.2 (1.1-34.7) months  - Group B: 5.0 (0.4-20.4) months  Local recurrence-free duration (LRFD) (range):  - Overall: 8.4 (0.4-34.7) months  - Group A: 19.6 (1.1-34.7) months  - Group B: 3 (0.4-20.4) months |  |  | Any grade:  - Fatigue: 16/46 (34.8%)  - Focal neurological deficit: 11/46 (23.9%)  - Headache: 9/46 (19.6%)  - Seizures: 8/46 (17.4%) |
| 14 | Dagogo-Jack et al., 2017 | Retrospective review of database | 89 patients, 36/89 MBM  - Median (range) age: 63 (18-88)  - Male/female (*N*): 59/30 | Systemic therapy:  - IPI: 47/89 (52.8)  - BRAF and/or MEKi: 15/89 (16.9)  Prior treatment for MBM:  - SRS: 13/36 (36.1)  - WBRT: 1/36 (2.8)  - Surgery: 2/36 (5.6)  - Surgery + radiation: 9/36 (25.0) | Pembrolizumab | Patients died during FU 36/89 (40.4);  16/36 (44.4) in MBM-patients  Median (90% CI) overall OS (all patients): 20.4 (15-N/A) months |  |  | Median time (*N*) to progression at any site:  - No BM: 6 months (53/89)  - Treated BM: 5 months (26/89)  - Untreated BM: 1.2 months (10/89) |  |
| 15 | Davies et al., 2011 | Retrospective review of database | 330 MBM-patients  - 303/330 ≤ 65 years | Systemic therapy: 307/330 (93.0) | TMZ and unknown other systemic therapies | Median survival from time of MBM-diagnosis: 4.65 months  Median OS with initially TMZ (7/330): 4.70 months  Median OS with other initial systemic therapies: 4.65 months |  |  |  |  |
| 16 | Davies et al., 2017 | Open-label, multi-cohort phase 2 trial | 125 MBM-patients  *Cohort A (BRAFV600E-mutant, asymptomatic MBM, without prior local brain-directed Tx):*  - Median (range) age: 52.0 (23-84)  - Male/female (*N*): 40/36  *Cohort B (BRAFV600E-mutant, asymptomatic MBM, with prior local Tx):*  - Median (range) age: 54.5 (36-74)  - Male/female (*N*): 10/6  *Cohort C (BRAFV600D/K/R-mutant, asymptomatic MBM, with or without prior local Tx):*  - Median (range) age: 63.0 (44-84)  - Male/female (*N*): 11/5  *Cohort D (BRAFV600D/E/K/R-mutant, symptomatic MBM, with or without prior local Tx):*  - Median (mean) age: 46.0 (23-68)  - Male/female (*N*): 11/6 | Steroid therapy:  - Cohort A: 3/76 (4)  - Cohort B: 1/16 (6)  - Cohort C: 0/16 (0)  - Cohort D: 5/17 (29) | Dabrafenib plus trametinib | Median (95% CI) OS:  - Cohort A: 10.8 (8.7-19.6) months  - Cohort B: 24.3 (7.9-NE) months  - Cohort C: 10.1 (4.6-17.6) months  - Cohort D: 11.5 (6.8-22.4) months  6-month OS rate (95% CI):  - Cohort A: 79 (68-87) %  - Cohort B: 81 (52-94) %  - Cohort C: 69 (40-86) %  - Cohort D: 88 (61-97) %  12-month OS rate (95% CI): - Cohort A: 46 (33-58) %  - Cohort B: 69 (40-86) %  - Cohort C: 44 (20-66) %  - Cohort D: 44 (20-66) % | Intracranial response:  - Cohort A: 44/76 (58%)  - Cohort B: 9/16 (56%)  - Cohort C: 7/16 (44%)  - Cohort D: 10/17 (59%) | Overall response:  - Cohort A: 44/76 (58%)  - Cohort B: 9/16 (56%)  - Cohort C: 7/16 (44%)  - Cohort D: 11/17 (65%) | Median (95% CI) PFS:  - Cohort A: 5.6 (5.3-7.4) months  - Cohort B: 7.2 (4.7-14.6) months  - Cohort C: 4.2 (1.7-6.5) months  - Cohort D: 5.5 (2.8-7.3)) months | Total grade 3/4 AE’s: 60/125 (48%) |
| 17 | Devito et al., 2011 | Retrospective review of database | 29 MBM-patients  - Median (range) age: 58 (30-83)  - Male/female (*N*): 17/12 | Other CT: 9 patients before TMZ, other patients after TMZ | WBRT combined with TMZ | Median (95% CI) OS from date of MBM: 44.4 (30.6-74.7) weeks  WBRT alone: 16 weeks |  |  | PFS (95% CI): 20.4 (16.6-30.9) weeks | - Thrombocytopenia grade 3: 3/29 (13.8%)  - Fatigue Grade 3: 1/29 (3.4%)  - Neutropenia Grade 3: 1/29 (3.4%)  - Lymphedema Grade 3: 1/29 (3.4%) |
| 18 | Diao et al., 2018 | Retrospective review of database | 72 MBM-patients  - Median age: 61  - Male/female (*N*): 49/23 | WBRT: 6/72 (8.3) | SRS combined with IPI |  | Local failure:  - SRS: 19%  - Concurrent IPI: 10%  - Nonconcurrent IPI: 19% |  |  |  |
| 19 | Diao et al., 2018 | Retrospective review of database | 91 MBM-patients  - Median (range) age: 62 (27-85)  - Male/female (*N*): 62/29 | - Surgery: 34/91 (37.4)  - WBRT: 7/91 (7.7)  - CT: 30/91 (33.0)  - TT: 41/91 (45.1) | SRS combined with IPI | Median OS: 10.6 months  Median OS:  - SRS: 7.8 months  - SRS + IPI: 15.1 months  *P*=0.02  Median OS for SRS + IPI:  - Nonconcurrent: 18.7 months  - Concurrent: 11.8 months  1-year OS:  - Nonconcurrent IPI (*N*=28): 63%  - Concurrent IPI (*N*=23): 50%  - No IPI: 28%  *P*=0.02 |  |  | 1-year FFS:  - No IPI: 10%  - Concurrent IPI: 9%  - Nonconcurrent IPI: 29% | Grade 3/4 AE’s:  - No IPI: 1/40 (2.5%)  - Concurrent: 2/23 (8.7%)  - Nonconcurrent:2 /28 (7.1%) |
| 20 | Di Giacomo et al., 2012 | Open-label, single-arm phase 2 trial | 86 patients, 20/86 MBM  - Median (range) age: 54 (43-66)  - Male/female (*N*): 60/26 | - Systemic for metastases: 42/86 (48.8)  - SRS for BM: 4/20 (20.0)  - WBRT for BM: 3/20 (15.0) | IPI combined with fotemustine | Median (95% CI) OS: 13.3 (8.9-19.9) months  Median (95% CI) OS MBM: 13.4 (10.0-N/A) months | Disease control whole group: 40/86 (46.5%, 95% CI: 35.7-57.6)  MBM separate (10/20): 50%, 95% CI: 27.2-72.8 |  | Median (95% CI) immune-related PFS:  - All patients: 5.3 (3.4-7.1) months  - MBM: 4.5 (0.3-8.7) months | Grade 3/4 treatment-related AE’s: 47/86 (54.7%) |
| 21 | Di Giacomo et al., 2014 | Phase 2 study | 86 patients, 20/86 MBM  - Median (range) age: 55 (43-66)  - Male/female (*N*): 60/26 | - Systemic for metastases: 42/86 (48.8)  - SRS for BM: 4/20 (20.0)  - WBRT for BM: 3/20 (15.0) | IPI combined with fotemustine | Median (95% CI) OS: 12.9 (7.1-18.7) months  Median (95% CI) OS MBM: 12.7 (2.7-22.7) months |  |  |  |  |
| 22 | Drago et al., 2018 | Retrospective review of database | 65 MBM-patients:  - Median (range) age: 49.2 (16.2-72.2)  - Male/female (*N*): 38/27 | - Surgery: 18/65 (27.7)  - SRS: 22/65 (33.8)  - WBRT: 20/65 (33.3)  - CT: 3/65 (4.6)  - Anti-PD1: 18 (27.7)  - Anti-CTLA-4: 12 (18.5) | BRAF/MEKi | Median (95% CI) OS: 9.5 (7.7-13.5) months |  |  | Median (95% CI) PFS: 5.3 (3.6-6.1) months |  |
| 23 | Du Four et al., 2018 | Retrospective review of database | 43 MBM-patients  - Median (range) age: 50 (33-84)  - Male/female (*N*): 14/29 | - SRS: 31/43 (72.1)  - WBRT: 8/43 (18.6)  - RT before Pembrolizumab: 28/40 (71.7)  - IPI: 29/43 (67.4) | Pembrolizumab | Median OS from Pembro: 11.3 months  Median OS from RT: 15.7 months |  |  |  |  |
| 24 | Dummer et al., 2013 | Open-label phase 1 trial | 24 MBM-patients  - Median (range) age: 47 (24-70)  - Male/female (*N*): 13/11 | - WBRT: 14/24 (58.3)  - SRS: 6/24 (25.0)  - Surgery: 4/24 (16.7)  - Surgery + RT: 4/24 (16.7) | Vemurafenib | Median (95% CI) OS: 5.3 (3.9-6.6) months | Overall partial response (PR) (both intra- and extracranial): 10/24 patients (42%, 95% CI: 22.1-63.4) |  | Median (95% CI) PFS: 3.9 (3.0-5.5) months |  |
| 25 | Dzienis et al., 2014 | Retrospective review of database | 22 MBM-patients  - Median (range) age: 49 (21-76)  - Male/female (*N*): 13/9 | - Surgery: 1/22 (4.5)  - WBRT: 5/22 (22.7)  - Surgery + WBRT: 4/22 (18.2)  - CT: 2/22 (9.1) | Vemurafenib | Median OS: 46 weeks in responding patients and  21 weeks in nonresponding patients |  |  | Median (range) time to CNS progression:  23 (12-60) weeks in responding patients and  14 (3-22) weeks in nonresponding patients |  |
| 26 | Falchook et al., 2012 | Phase 1 dose-escalation trial | 46 patients, 10 MBM-patients  - Median (range) age non-MBM: 54.5 (41-60)  - Median (range) age MBM: 62.5 (29-65)  - Male/female non-MBM (*N*): 21/15  - Male/female MBM (*N*): 6/4 | Unknown | Dabrafenib |  |  |  | Median (95% CI) PFS MBM: 4.2 (3.3-5.3) months |  |
| 27 | Fang et al., 2017 | Retrospective review of database | 137 MBM-patients  - Median (range) age: 57 (16-83)  - Male/female (*N*): 94/43 | - WBRT: 56/137 (40.9)  - CT: 86/137 (62.8) | SRS combined with CTLA-4 blockade and PD-1 inhibition | Median OS: 16.9 months |  |  |  |  |
| 28 | Fang et al., 2017 | Retrospective review of database | 235 MBM-patients:  - Median (range) age: 56 (4-88)  - Male/female (*N*): 157/78 | - Systemic Tx: 136/235 (58) | - SRS/Surgery: 159/235 (67%)  - Local ablative + WBRT: 17/235 (7%)  - WBRT: 39/235 (17%)  - Systemic: 20/235 (9%) | Median (95% CI) OS for all patients: 11.3 (9-13) months |  |  |  |  |
| 29 | Foppen et al., 2017 | Retrospective review of database | 146 MBM-patients:  - Median (range) age: 54 (23-80)  - Male/female (*N*): 80/66 | - SRS during TT: 18/146 (12)  - WBRT during TT: 26/146 (18)  - Tx after progression on TT: 38/146 (26) | Vemurafenib (58%), dabrafenib (21%) or dabrafenib + trametinib (21%) | Median (95% CI) OS: 6.6 (5.7-7.4) months  Median (95% CI) OS:  - Vemurafenib: 5.7 (4.6-6.8) months  - Dabrafenib: 8.8 (3.9-13.7) months  - Dabrafenib + trametinib: 11.2 (6.8-15.7) months | Intracranial control rate:  - Total: 99/146 (68%)  - Vemurafenib: 60/85 (71%)  - Dabrafenib: 21/31 (68%)  - Dabrafenib + trametinib: 18/30 (60%) |  | Median (95% CI) intracranial PFS: 4.1 (3.2-5.0) months  Median (95% CI) intracranial PFS:  - Vemurafenib: 3.6 (3.5-3.8) months  - Dabrafenib: 5.7 (3.0-8.4) months  - Dabrafenib + trametinib: 5.8 (3.2-8.5) months  Median (95% CI) extracranial PFS: 4.6 (3.4-5.9) months  Median (95% CI) extracranial PFS:  - Vemurafenib: 4.0 (3.3-4.7) months  - Dabrafenib: 5.8 (3.3-8.3) months  - Dabrafenib + trametinib: 7.3 (3.9-10.8) months |  |
| 30 | Gabani et al., 2018 | Retrospective review of database | 1104 MBM-patients:  - Median (range) age: 62.0 (18-90)  - Male/female (*N*): 797/307 | Unknown | RT combined with IT (mostly IPI):  - WBRT: 638/1104 (57.8%) - WBRT + IT: 103/1104 (9.3%)  - SRS: 274/1104 (24.8%)  - SRS + IT: 89/1104 (8.1%) | Median (95% CI) OS:  - RT: 6.2 (5.6-6.8)  - RT + IT: 11.1 (8.9-13.4)  *P*<0.001  Median (95% CI) OS:  - SRS: 11.9 (9.8-14.0)  - SRS + IT: 17.0 (10.7-23.2)  - WBRT: 4.4 (3.9-4.9)  - WBRT + IT: 8.5 (6.5-10.5)  *P*<0.001 |  |  |  |  |
| 31 | Gaudy-Marqueste et al., 2014 | Retrospective review of database | 30 MBM-patients, 24/30 GKRS under BRAFi, 6/30 GKRS before BRAFi  *GKRS under BRAFi*  - Median age 50 (24-68)  - Male/female (*N*): 13/11  *GKRS before BRAFi*  - Median (range) age: 61.2 (43-78)  - Male/female (*N*): 3/3 | Unknown | GKRS and BRAFi (26/30 Vemurafenib, 4/30 Dabrafenib) | Median (95% CI) OS from first GKRS under BRAFi: 24.8 (10.1-39.6) weeks  Median (95% CI) OS from first dose BRAFi: 48.85 (30.62-67.08) weeks  6-months survival: 78.8% |  |  | Median time to new MBM from first GKRS under BRAFi  was 12.85 weeks (95% CI: 11.6–14.07) |  |
| 32 | Gaudy-Marqueste et al., 2017 | Retrospective review of database | 179 MBM-patients  - Median age: 59.3  - Male/female (*N*): 96/83  *BRAF-mutated:*  - TT or IT after SRS: 76/105  - No TT or IT after SRS: 29/105  *BRAF-wild-type:*  - TT or IT after SRS: 32/68  - No TT or IT after SRS: 36/68 | *BRAF-mutated:*  - TT or IT after SRS: 28/76 (36.8)  - No TT or IT after SRS: 10/29 (34.5)  *BRAF-wild-type:*  - TT or IT after SRS: 7/32 (21.9)  - No TT or IT after SRS: 6/36 (16.7) | SRS combined with IPI, anti-PD1, BRAFi and BRAF/MEKi | Median OS:  - SRS: 2.29 months  - SRS + IT or TT: 10.95 months  Median OS (months):  *BRAF-mutated:*  - No Tx: 2.62  - BRAF±MEKi: 7.31  - BRAF±MEKi + IT: 14.82  *BRAF-wild-type:*  - No IT: 2.29  - Anti-PD1: 12.26  - IPI: 8.62  - IPI + anti-PD1: 14.07  *BRAF-mutated treated with BRAF±MEKi:*  - Median OS: 7.31 months  - 1-year OS rate: 35.3%  - 2-year OS rate: 8.8%  *BRAF-wild-type treated with anti-PD1*:  - Median OS: 12.26 months  - 1-year OS rate: 63.6%  - 2-year OS rate: 23.9% |  |  |  |  |
| 33 | Gerber et al., 2014 | Retrospective review of database | 13 MBM-patients  - Median (range) age: 64 (36-85)  - Male/female (*N*): 7/6 | - Systemic: 8/13 (61.5)  - RT: 5/13 (38.5)  - Craniotomy: 5/13 (38.5) | WBRT combined with IPI | Median OS: 4 months  1-year survival rate: 15.4% |  |  |  | Grade 1-2 fatigue/nausea/cognitive changes: 54%/23%/23%  -Grade 3 cognitive changes: 1/13 (8%)  Intra-tumoral haemorrhage: 10/10 with follow-up imaging |
| 34 | Gibney et al., 2015 | Retrospective chart review | 283 MBM-patients  - Mean (SD) age: 57.2 ± 11.5  - Male/female (*N*): 172/111 | - Systemic: 125/283 (44.2)  - SRS: 48/283 (17.0)  - WBRT: 34/283 (12.0)  - Surgery: 30/283 (10.6) | Vemurafenib | 6- and 12-month OS rate (95% CI): 85.6% (80.1-89.8)/59.0% (49.5-67.2)  With prior local treatments:  - 6-month OS rate (95% CI): 81.8% (70.9-88.9)  - 12-month OS rate (95% CI): 61.4% (45.9-73.6)  Without prior local treatments:  - 6-month OS rate (95% CI: 87.9% (81.0-92.3)  - 12-month OS rate (95% CI): 59.4% (47.7-69.3) | Intracranial response: 136/283 (48.1%) | Extracranial response: 129/283 (45.6%) |  |  |
| 35 | Goldberg et al., 2016 | Phase 2 trial | 18 MBM-patients  - Median (range) age: 64 (41-85)  - Male/female (*N*): 12/6 | - Systemic: 14/18 (77.8)  - Resection: 8/18 (44.4)  - WBRT: 3/18 (16.7)  - SRS: 9/18 (50.0) | Pembrolizumab | Median OS: not reached | Brain metastasis response rate: 22% |  |  | Pembrolizumab was safe and well-tolerated  - Grade 1-2 headache/dizziness/stroke/seizure: 17%/6%/0%/17%  - Grade 3: cognitive changes/elevated aminotransferases: both 6% |
| 36 | Gorka et al., 2017 | Retrospective review of database | 30 MBM-patients  - Median (range) age: 59.2 (21.8-75.1)  - Male/female (*N*): 15/15 | - Surgical resection: 9/30 (30)  - SRS: 10/30 (33)  - WBRT: 16/30 (53)  - CT: 6/30 (20)  - Vemurafenib: 2/30 (7) | Dabrafenib + control group | Median OS: 8.8 months |  |  | Median PFS: 5.5 months | Any, grade 3/4: 3/30 (10%)  - Rash: 5/30 (17%) |
| 37 | Gummadi et al., 2014 | Retrospective chart review | 191/436 MBM-patients  - Median (range) age: 59.1 (24-96)  - Male/female (*N*): 258/178 | Unknown | Vemurafenib |  |  | Lower incidence of BM in patients with BRAF-mutated tumors who took Vemurafenib before the development of BM vs. those who did not: incidence rate ratio (95% CI): 0.51 (0.30-0.86) (*P*=0.009) |  |  |
| 38 | Gupta et al., 2016 | Phase 2 trial | 24 MBM-patients  *WBRT + Vandetanib 10/24*  - Mean (range) age: 57 (42-77)  Male/female (*N*): 5/5  *WBRT + placebo* *8/24*  - Mean (range) age: 64 (44-83)  - Male/female (*N*): 3/5  *Safety cohort 6/24*  - Mean (range) age: 69 (55-77)  - Male/female (*N*): 4/2 | *WBRT + Vandetanib:*  - Surgery: 10/10 (100)  - Medical: 6/10 (60.0)  - RT: 3/10 (30.0)  *WBRT + placebo:*  - Surgery: 7/8 (87.5)  - Medical: 6/8 (75.0)  - RT: 1/8 (12.5)  *Safety cohort:*  - Surgery: 6/6 (100)  - Medical: 2/6 (33.3)  - RT: 2/6 (33.3) | WBRT versus WBRT combined with Vandetanib | Median (90% CI) OS WBRT + Vandetanib: 4.6 (1.6-6.3) months  Median (90% CI) OS WBRT + placebo: 2.5 (0.2-7.2) months  (*P*=0.54) |  |  | Median (90% CI) PFS: - Vandetanib: 3.3 (1.6-6.5) months  - Placebo: 2.5 (0.2-4.8) months  (*P*=0.34) | *WBRT + Vandetanib + safety cohort:*  - Fatigue 6/16 (37.5%)  - Nausea 4/16 (25/0%)  - Headache 3/17 (17.6%)  - Confusion 4/16 (25.0%)  - Alopecia 5/16 (31.3%)  - Any grade 3: 50%  *WBRT + placebo:* no AEs |
| 39 | Harding et al., 2015 | Retrospective review of database | 27 MBM-patients  - Median (range) age: 53 (25-86)  - Male/female (*N*): 15/12 | - WBRT: 9/27 (33.3)  - SRS: 1/27 (3.7)  - Craniotomy 1/27 (3.7)  - Multimodality: 2/27 (7.4)  - IPI: 9/27 (33.3) | Vemurafenib | Median (95% CI) OS: 7.5 (4.3-not reached) months  1-year (95%CI) OS: 30.4% (13.2-50.1) |  |  | Median (95% CI) PFS: 4.1 (2.7-7.9) months | WBRT + Vemurafenib:  - grade 2 radiation dermatitis: 3/10 (30.0%)  - Painful cutaneous reaction: 1/10 (10.0%)  Overall, grade 3 or more:  - Rash: 6/27 (22.2%)  - Hyperproliferative skin lesions: 4/27 (14.8%)  - Photosensitivity: 3/27 (11.1%)  - Seizure: 1/27 (3.7%) |
| 40 | Haueis et al., 2016 | Retrospective review of database | 96 MBM-patients  *BRAFi group* (targeted therapy) (42/96):  - Median (range) age: 54 (24-73)  - Male/female (*N*): 25/17  *Control group* (no targeted therapy) (54/96):  - Median (range) age: 65 (35-86)  - Male/female (*N*): 36/18 | *BRAFi group:*  - Targeted therapy: 32/42 (76.2)  - Systemic therapies: 10/42 (23.8)  *Control group:*  - Systemic therapies: 20/54 (37.0) | BRAFi and control group  *Control group:*  Systemic therapy: 48/54 (89.0):  - Chemo: 21/54 (38.9)  - Immuno: 13/54 (24.1)  - Multikinase inhibitor: 1/54 (1.85)  - Chemo + immune: 7/54 (13.0)  - Chemo and multikniase inhibitor: 6/54 (11.1) |  |  | BRAFi group: significantly greater increase in number of BM  BRAFi group: mean size of new lesions was smaller |  |  |
| 41 | Hofmann et al., 2011 | Prospective phase 3 study | 117 metastatic melanoma patients  *34/117 BSC*  - Median (range) age: 66 (39-75)  - Male/female (*N*): 18/16  - MBM 8/34 (23.5)  *83/117 BSC + CVD*  - Median (range) age: 62 (33-77)  - Male/female (*N*): 47/36  - MBM 7/83 (8.4) | *BSC:*  - CT: 26/34 (76.5)  - Immuno: 4/34 (11.8)  - Chemo-immuno: 7/34 (20.6)  *BSC + CVD:*  - CT: 52/83 (62.7)  - Immuno: 15/53 (28.3)  - Chemo-immuno: 33/83 (39.8) | Best supportive care (BSC) alone and BSC combined with Cisplatin, Vindesine and Dacabazine (CVD) | OS multivariate Cox, OR (95% CI) for MBM: 2.74 (1.52-4.94)  Median OS:  Cohort 1: 4.6 months  Cohort 2: 7.6 months  (*P*=0.014, *P*< 0.001 for MBM) |  |  |  | BSC: 1 grade IV event  BSC + CVD: 14 grade IV events |
| 42 | Hong et al., 2010 | Retrospective review of database | 26 MBM-patients  *17/26 ACT with TIL*  - Mean (range) age: 41 (17-61)  - Male/female (*N*): 9/8  *9/26 TCR-transduced lymphocytes*  - Mean (range) age: 39 (25-56)  - Male/female (*N*): 3/6 | Unknown | ACT (cyclophosphamide and fludarabine with or without total body irradiation), followed by the infusion of autologous TIL or autologous peripheral blood lymphocytes | *ACT+TIL:*  - Median OS: 8.5 months  - 2-year survival: 40%  *TCR-lymphocytes:*  - Median OS: 15 months | *ACT+TIL:*  - Complete response in the brain: 7/17 (41.2%)  - Overall partial response: 6/17 (35.3%)  *ACT+TCR-lymphocytes:*  - Complete response in the brain: 2/9 (22.2%)  - Overall partial response 1/2 (50.0%) |  |  | *ACT+TIL:*  - Subarachnoid haemorrhage: 1/17 (5.9%) |
| 43 | Iorgulescu et al., 2018 | Retrospective review of database | 2753 MBM-patients | Unknown | Checkpoint blockade IT (CBI) and BRAFV600-TT | 4-year OS:  - Before FDA approvals for CBI and BRAFV600-TT: 7.4%  After approvals: 14.1%  *P*<0.001  Median (95% CI) OS:  - No initial CBI: 5.2 (4.7-5.9) months  - Initial CBI: 12.4 (10.4-15.8) months (*P*<0.001) |  |  |  |  |
| 44 | Jones et al., 2015 | Retrospective chart review | 12 MBM-patients  - Median (range) age: 59.2 (39-72)  - Male/female (*N*): 8/4 | - SRS: 10/12 (83.3)  - WBRT: 5/12 (41.7) | IPI combined with craniotomy | Median OS: 7 months |  |  |  |  |
| 45 | Kaidar-Person et al., 2017 | Retrospective review of database | 58 MBM-patients  *29/58 Non-immunoTx:*  - Mean age: 62  *29/58 ImmunoTx:*  - Mean age: 57 | *Non-immunoTx:*  - WBRT: 3/29 (10.3)  *ImmunoTx:*  - WBRT: 4/29 (13.8) | SRS and SRS combined with immunotherapy | Median (95% CI) OS:  *-* Non-immunoTx: 5.5 (3.8-8.4) months  *-* ImmunoTx: 15 (10-15.8) months  (*P*=0.0013) | Local intracranial failure:  - Non-immunoTx:  4/29 (13.8%)  - ImmunoTx: 14/29 (48.3%) | Distant intracranial failure:  - Non-immunoTx: 13/29 (44.8%)  - ImmunoTx: 19/29 (65.5%) | Failure Free Survival (FFS):  - Non-immunoTx: 4.3 months  - ImmunoTx:  3.5 months | Radiation necrosis:  - Non-immunoTx: 0  - ImmunoTx: 8/29 (27.6%)  Hemorrhage:  - Non-immunoTx: 2/29 (6.9%)  - ImmunoTx: 7/29 (24.1%)  Any brain toxicity:  - Non-immunoTx vs. ImmunoTx: HR: 9.6 (95% CI 2.1-42.3)  (*P*=0.0029) |
| 46 | Kiess et al., 2015 | Retrospective review of database | 46 MBM-patients  - Median (range) age: 57 (24-76)  - Male/female (*N*): 27/19 | - TMZ: 21/46 (45.7)  - IL-2: 7/46 (15.2)  - Cisplatin, Vinblastine and TMZ: 17/46 (37.0) | SRS combined with IPI | *SRS during IPI (N*=15*):*  - 1-year OS: 65%  *SRS before IPI (N*=19*):*  - 1-year OS: 56%  *SRS after IPI (N*=12*):*  - 1-year OS: 40%  (*P*=0.008) | *SRS during IPI:*  - 1-year local recurrence: 0%  *SRS before IPI:*  - 1-year local recurrence 13%  *SRS after IPI:*  - 1-year local recurrence: 11%  (*P*=0.21) |  |  | Grade 3 to 4 toxicities: 9/46 (19.6%) |
| 47 | Kluger et al., 2018 | Phase 2 trial | 23 MBM-patients:  - Median (range) age: 65 (40-84)  - Male/female (*N*): 15/8 | - Surgical resection: 12/23 (52)  - WBRT: 5/23 (22)  - SRS: 12/23 (52)  - IPI: 13/23 (57)  - BRAFi: 4/23 (17) | Pembrolizumab | Median (95% CI) OS: 17 (10-NR) months |  |  | Median (95% CI) PFS: 2 (2-NR) months | Grade 3/4 AE’s:  - Cognitive dysfunction: 1/23 (4%)  - Rash: 1/23 (4%)  - Acidosis: 1/23 (4%)  - Hyponatremia: 1/23 (4%)  - Elevated transaminases: 1/23 (4%) |
| 48 | Knisely et al., 2012 | Retrospective chart review | 77 MBM-patients  - Median (range) age: 61 (24-89)  - Male/female (*N*): 49/28  *IPI: 27/77:*  - IPI before SRS: 11/27 (40.7)  - IPI after SRS: 16/27 (59.3) | - CT: 48/77 (62.3)  - TT: 14/77 (18.2)  - WBRT: 27/77 (35.1) | SRS combined with IPI | *Entire cohort:*  - Median (95% CI) OS: 8.8 (4.93-18.4) months  *IPI group:*  - Median (95% CI) OS: 21.3 (6.43-26.7) months  - 2-year (95% CI) OS: 47.2% (24.8-66.8)  *Non-IPI group:*  - Median (95% CI) OS: 4.9 (3.3-10.4) months  - 2-year (95% CI) OS: 19.7% (9.0-33.5)  *IPI before SRS:*  - Median (95% CI) OS: 19.8 (1.5-not reached) months  *IPI after SRS:*  - Median (95% CI) OS: 21.3 (15.7-not reached) months  (*P*=0.58) |  |  |  |  |
| 49 | Konstantinou et al., 2014 | Retrospective review of database | 38 MBM-patients  - Male/female (*N*): 26/12 | - Surgical resection: 8/38 (21.1)  - SRS: 7/38 (18.4) - WBRT: 3/38 (7.9) | - IPI: 24/38 (63.2)  - IPI + RT: 6/38 (15.8)  - IPI + surgery: 4/38 (10.5)  - IPI + RT + surgery: 4/38 (10.5) | Median (range) OS all patients: 101 days (54-154)  1-year OS: 10.5% | BM-control rate: 6/38 (16%) |  |  | - Drug-related AE: 26/38 (68.4%)  - GI: 15/38 (39.5%)  - Skin: 6/38 (15.8%)  - Intracranial haemorrhage: 8/38 (21.1%) |
| 50 | Kotecha et al., 2018 | Retrospective review of database | 366 MBM-patients:  - Mean (SD) age:57 (14)  - Male/female (*N*): 227/139  - BRAF-mutated: 35/366 (9.6%)  - BRAF-wild-type: 43/366 (11.7%)  - BRAF unknown: 288/366 (78.7%) | - Surgery: 94/366 (26)  - WBRT: 217/366 (59)  - SRS: 182/366 (50) | BRAFi, anti-PD1, anti-CTLA-4, Cytokines (± SRS: 191/366, 52%) | Median (95% CI) OS:  - Overall: 6 months  - BRAF+: 9 (6-13) months  - BRAF-WT: 5 (4-7) months  - BRAF unknown: 6 (5-7) months  6-months OS (range):  - Overall: 50% (45-55%)  - BRAF+: 67% (50-81%)  - BRAF-WT: 44% (30-59%)  - BRAF unknown: 49% (44-55%)  12-months OS (range):  - Overall: 24% (20-29%)  - BRAF+: 35% (20-53%)  - BRAF-WT: 28% (16-43%)  - BRAF unknown: 23% (18-28%) | 12-months (95% CI) intracranial failure:  - BRAF+: 58% (41-76%)  - BRAF-WT: 43% (28-59%)  - BRAF unknown: 42% (36-48%)  SRS + systemic therapy, local failure (95% CI):  - 6-months: 12% (10-14%)  - 12-months: 14% (11-16%)  - 12-months BRAF+: 6% (2-11%)  - 12-months BRAF-WT: 22% (13-32%)  *P*<0.01 | Distant failure (95% CI):  - 6-months SRS alone: 48% (39-58%)  - 12-months SRS alone: 69% (59-78%)  - 12-months BRAF+: 69% (49-84%)  - 12-months BRAF-WT: 72% (50-87%)  12-months distant failure BRAF+:  - No BRAFi: 95%  - BRAFi: 68%  *P*=0.03 |  |  |
| 51 | Ladwa et al., 2018 | Retrospective review of database | 142 MBM-patients:  - Median (range) age: 57 (17-82)  - Male/female (*N*): 82/60 | Unknown | - SRS: 34/142 (23.9%)  - WBRT: 52/142 (36.6%)  - IPI: 35/142 (24.6%)  - Pembrolizumab: 36/142 (25.4%)  - Nivolumab: 19/142 (13.4%)  - IPI+Nivolumab: 8/142 (5.6%) | Median (95% CI) OS: - Overall: 8 (6.9-9.1) months  - IPI: 10 (4.5-15.5) months  - Anti-PD1: 9 (3.1-14.9) months | Intracranial response rate (complete and partial) for IT (*N*=65): 34% |  |  |  |
| 52 | Long et al., 2012 | Phase 2 trial | 172 MBM-patients: 89/172 cohort A, 83/172 cohort B  *Cohort A (no previous local treatment):*  - Median (range) age: 52 (43-63)  - Male/female (*N*): 65/24  *Cohort B (previous surgery, WBRT or SRS):*  - Median (range) age: 53 (44-62)  - Male/female (*N*): 55/28 | *Cohort A:*  - CT: 23/89 (25.8)  - IT: 5/89 (5.6)  - Small-molecule targeted: 1/89 (1.1)  - Biological: 1/89 (1.1)  *Cohort B:*  - CT: 35/83 (42.2)  - IT: 14/83 (16.9)  - Small-molecule targeted: 2/83 (2.4)  - Biological: 1/83 (1.2) | Dabrafenib | *Val600Glu BRAF mutant:*  - Median (95% CI) OS cohort A: 33.1 (25.6-NR) weeks  - Median (95% CI) OS cohort B: 31.4 (25.7-NR) weeks  *Val600Lys BRAF mutant:*  - Median (95% CI) OS cohort A: 16.3 (6.9-22.4) weeks  - Median (95% CI) OS cohort B: 21.9 (15.3-NR) weeks |  |  | *Val600Glu BRAF mutant:*  - Median (95% CI) PFS cohort A: 16.1 (15.7-21.9) weeks  - Median (95% CI) PFS cohort B: 16.6 (15.9-23.7) weeks  *Val600Lys BRAF mutant:*  - Median (95% CI) PFS cohort A: 8.1 (3.1-16.1) weeks  - Median (95% CI) PFS cohort B: 15.9 (7.9-22.4) weeks | - Grade 3 or worse AEs: 38/172 (22.1%)  - Intracranial haemorrhage: 10/172 (5.8%); 1 treatment-related |
| 53 | Long et al., 2018 | Phase 2 study | 79 MBM-patients  *Cohort A (nivolumab+IPI):*  - Median (range) age: 59 (53-68)  - Male/female (*N*): 29/6  *Cohort B (nivolumab):*  - Median (range) age: 63 (52-74)  - Male/female (*N*): 19/6  *Cohort C (nivolumab):*  - Median (range) age: 51 (48-56)  - Male/female (*N*): 11/5 | *Cohort A:*  - BRAF/MEKi: 8/35 (23)  - Surgery: 0/35 (0)  - SRS: 0/35 (0)  - WBRT: 0/35 (0)  *Cohort B:*  - BRAF/MEKi: 6/25 (24)  - Surgery: 0/25 (0)  - SRS: 0/25 (0)  - WBRT: 0/25 (0)  *Cohort C:*  - BRAF/MEKi: 12/16 (75)  - Surgery: 9/16 (56)  - SRS: 8/16 (50)  - WBRT: 7/16 (44) | Nivolumab + IPI vs. nivolumab alone | Median (95% CI) OS:  - Cohort A: NR (8.5-NR)  - Cohort B: 18.5 (6.9-NR) months  - Cohort C: 5.1 (1.8-NR) months  6-months (95% CI) OS:  - Cohort A: 78% (65-94%)  - Cohort B: 68% (52-89%)  - Cohort C: 44% (25-76%) | Intracranial response, overall (95% CI):  - Cohort A: 16/35 (29-63)  - Cohort B: 5/25 (7-41)  - Cohort C: 1/16 (0-30) | Extracranial response, overall (95% CI):  - Cohort A: 17/35 (37-75)  - Cohort B: 6/25 (11-52)  - Cohort C: 3/16 | Median (95% CI) intracranial PFS:  - Cohort A: NR (2.9-NR)  - Cohort B: 2.5 (1.7-2.8) months  - Cohort C: 2.3 (1.4-4.3) months  Median (95% CI) extracranial PFS:  - Cohort A: 13.8 (4.9-NR) months  - Cohort B: 2.6 (1.8-13.8) months  - Cohort C: 2.6 (2.1-13.6) months | *Cohort A:*  - Grade 3: 19/35 (54%)  - Grade 4: 3/35 (9%)  *Cohort B:*  - Grade 3: 4/25 (16%)  *Cohort C:*  - Grade 3: 2/16 (13%) |
| 54 | Lonser et al., 2011 | Retrospective review of database | 41 MBM-patients  - Median (range) age: 44.4 (19.2-63.1)  - Male/female (*N*): 24/17 | WBRT: 15/41 (36.6) | Surgical resection combined with immunotherapy  *Immunotherapy:*  - IL-2: 35/41 (85.4)  - Vaccine: 5/41 (12.2)  - MAb: 1/41 (2.4) | Median (range) OS: 16.1 (2.3-95.2) months  Median (range) OS with adjuvant WBRT: 19.1 (4.2-64.7) months  Median (range) OS without adjuvant WBRT: 12.2 (2.3-95.2) months  (*P*=0.65)  Response to systemic immunoTx:  - Mean (range) OS: 33.8 (3.5-75.9) months  - 6-month survival rate: 88.2%  - 12-month survival rate: 70.6%  No response to systemic immunoTx:  - Mean (range) OS: 16.6 (2.3-95.2) months (*P*=0.02)  - 6-month survival rate: 62.5% (*P*=0.09)  - 12-month survival rate: 37.5% (*P*=0.06) | Overall LC-rate: 92.5%  Median (range) time to local recurrence:  - WBRT: 16.4 (2.7-64.6) months  - Non-WBRT: 10.2 (1.5-95.0) months | Distant recurrence rate: 37.7% |  |  |
| 55 | Ly et al., 2015 | Retrospective review of database | 52 MBM-patients  - Median (range) age: 52 (19-64)  - Male/female (*N*): 40/12  BRAF mutation: 31/52 (59.6)  BRAFi: 17/31 (54.8) | Systemic: median 2 | SRS with or without treatment for BRAF-mutation | 1-year median OS for all patients: 12 months  1-year OS rate: 48.0%  *BRAF mutation:*  - 1-year OS rate: 50.0%  *No BRAF mutation:*  - 1-year OS rate: 45.0%  (*P*=0.30)  *BRAFi:*  - 1-year OS rate: 50.2%  *No BRAFi:*  - 1-year OS rate: 42.9%  (*P*=0.82) | *Entire cohort:*  - 1-year LC-rate: 69.2%  *BRAF mutation:*  - BRAFi: 1-year LC-rate:85.0%  - No BRAFi: 1-year LC-rate: 51.5%  (*P*=0.0077) | *Entire cohort:*  - 1-year DC-rate: 33.0%  - Median time to failure: 5.5 months  *BRAF mutation:*  - BRAFi: 1-year DC-rate:32.3%  - No BRAFi: 1-year LC-rate: 33.0% |  | - Intratumoral haemorrhage: 29.2% of the lesions after SRS |
| 56 | Mangana et al., 2017 | Retrospective review of database | 395 stage IV melanoma patients:  - Median (range) age: 57 (13.6-88.5)  - Male/female (*N*): 239/156  - MBM: 171/395 | CT: 112/395 (28.4) | IT, TT (BRAF±MEKi), CT | Median OS MBM:  - Overall: 8.1 months  - CT (*N*=18): 6.1 months  - TT (*N*=23): 7.2 months  - IT (*N*=14): 10.9 months  - IT + TT (*N*=6): 9.1 months |  |  |  |  |
| 57 | Margolin et al., 2012 | Phase 2 trial | 72 MBM-patients; 51/72 cohort A, 21/72 cohort B  *Cohort A (neurologically asymptomatic and no corticosteroids):*  - Median (range) age: 59 (33-79)  - Male/female (*N*): 33/18  *Cohort B (neurologically symptomatic and corticosteroids):*  - Median (range) age: 57 (30-74)  - Male/female (*N*): 11/10 | *Cohort A:*  - IT: 24/51 (47.1)  - TT: 8/51 (15.7)  - CT: 22/51 (43.1)  - WBRT: 17/51 (33.3)  - SRS: 4/51 (7.8)  *Cohort B:*  - IT: 5/21 (23.8)  - TT: 3/21 (14.3)  - CT: 14/21 (66.7)  - WBRT: 5/21 (23.8)  - SRS: 0/21 (0) | IPI | *Cohort A:*  - Median (95% CI) OS: 7.0 (4.1-10.8) months  - 6-month (95% CI) OS: 55% (41-68)  - 12-month (95% CI) OS: 31% (18-44)  - 24-month (95% CI) OS: 26% (14-39)  *Cohort B:*  - Median (95% CI) OS: 3.7 (1.6-7.3) months  - 6-month (95% CI) OS: 38% (17-59)  - 12-month (95% CI) OS: 19% (2-36)  - 24-month (95% CI) OS: 10% (0-22) |  |  | *Cohort A:*  - Median (95% CI) PFS**: 1.4 (1.2-2.6)  *Cohort B:*  - Median (95% CI) PFS**: 1.2 (1.2-1.3) | *Cohort A, any grade:*  - Headache: 18/51 (35.3%)  - Nausea: 22/51 (43.1%)  - Diarrhoea: 25/51 (49.0%)  - Vomiting: 13/51 (25.5%)  *Cohort B, any grade:*  - Headache: 6/21 (28.6%)  - Nausea: 4/21 (19.0%)  - Diarrhoea: 9/21 (42.9%)  - Vomiting: 1/21 (4.8%) |
| 58 | Martin et al., 2018 | Retrospective review of database | 145 MBM-patients  *No IT:* 73/145  *IT:* 72/145 | No prior systemic Tx | SRS + IT (IPI, pembrolizumab, nivolumab) |  |  |  |  | IT and radiation necrosis: HR = 4.02 (95% CI 1.17-13.82), *P*=0.03  Radiation necrosis:  - IPI vs. no IT: HR = 4.70 (95% CI 1.36-16.19), *P*=0.01  - anti-PD1 vs. no IT: HR = 3.57 (95% CI 0.94-13.53), *P*=0.06 |
| 59 | Mastorakos et al., 2018 | Retrospective review of database | 198 MBM-patients:  *Group A (BRAFV600+, no BRAFi), n=23:*  - Median (range) age: 53 (20-80)  - Male/female (*N*): 12/11  *Group B (BRAFV600+, BRAFi), n=67:*  - Median (range) age: 47 (16-74)  - Male/female (*N*): 38/29  *Group C (BRAF-WT), n=108:*  - Median (range) age: 61 (13-84)  - Male/female (*N*): 84/24 | Unknown | SRS combined with BRAFi | Median (95% CI) OS after SRS:  - Cohort A: 36 (0-76.1) months  - Cohort B: 13 (9.4-16.7) months  - Cohort C: 7 (5.6-8.4) months  1-year survival rate after SRS:  - Cohort A: 34.8%  - Cohort B: 52.2%  - Cohort C: 28.7%  2-year survival rate after SRS:  - Cohort A: 30.4%  - Cohort B: 20.9%  - Cohort C: 8.3%  SRS before BRAFi better survival compared SRS after BRAFi, p<0.001 or concurrently to SRS, *P*=0.007 |  |  |  | Intracerebral hematoma:  - No BRAFi: 3%  - BRAFi: 10.4%  *P*=0.03 |
| 60 | Mathew et al., 2013 | Retrospective review of database | 58 MBM-patients; 25/58 SRS + IPI, 33/58 SRS  *SRS + IPI:*  - Median (range) age: 62 (27-87)  - Male/female (*N*): 13/12  *SRS:*  - Median (range) age: 57 (27-91)  - Male/female (*N*): 17/16 | *SRS+IPI:*  - CT: 12/25 (49.0)  *SRS:*  - CT: 13/33 (39.4) | SRS combined with IPI | *Entire cohort:*  - OS: 5.9 months  *SRS + IPI:*  - 6-month OS: 56%  *SRS:*  - 6-month OS: 45%  (*P*=0.18) | *Entire cohort:*  - Median LC: 8.7 months  - Local failure: 24/58 (41.4)  *SRS + IPI:*  - 6-month LC rate: 63%  - 6-month freedom of new MBM: 35%  *SRS:*  - 6-month LC rate: 65%  (*P*=0.55)  - 6-month freedom of new MBM: 47%  (*P*=0.48) |  |  | Intracranial haemorrhage:  - SRS + IPI: 7/25 (29.0%)  - SRS: 10/33 (30.3%) |
| 61 | McArthur et al., 2017 | Phase 2 study | 146 MBM-patients; 90/146 cohort 1, 56/146 cohort 2  *Cohort 1 (previously untreated BM):*  - Median (range) age: 55.5 (26-82)  - Male/female (*N*): 56/34  *Cohort 2 (previously treated BM):*  - Median (range) age: 52.5 (28-83)  - Male/female (*N*): 34/22 | *Cohort 1:*  - Systemic: 18/90 (20.0)  *Cohort 2:*  - Surgery: 34/56 (60.7)  - SRS: 28/56 (50.0)  - WBRT: 15/56 (26.8) | Vemurafenib | *Cohort 1:*  - Median (range) OS: 8.9 (0.6-34.5) months  *Cohort 2:*  - Median (range) OS: 9.6 (0.7-34.3) months | Median (range) time to development of new MBM:  *Cohort 1:* 14.9 (3.5-33.4) months  *Cohort 2:* 14.5 (2.8-27.4) months |  | *Cohort 1:*  - Median (range) PFS:3.7 (0.03-33.4) months  *Cohort 2:*  - Median (range) PFS: 4.0 (0.3-27.4) months | *Cohort 1:*  - Grade 3/4 AEs: 59/90 (65.6%)  *Cohort 2:*  - Grade 3/4 AEs: 36/56 (64.3%) |
| 62 | Menzies et al., 2015 | Retrospective review of database | 142 patients; 30/142 MBM-patients  *BRAFi: 111/142 patients; 30 MBM*  - Median (range) age: 49.3 (17-83)  - Male/female (*N*): 71/40  *BRAFi + MEKi:*  *31/142 patients; 0 MBM*  - Median (range) age: 50.4 (26-80)  - Male/female (*N*): 18/13 | No MAPKi or immunotherapy in the metastatic setting | BRAFi (Dabrafenib 70/111 (63.1) or  Vemurafenib 41/111 (36.9)) |  | Partial response: 22/30 (73.3%) |  |  |  |
| 63 | Milsch et al., 2018 | Retrospective review of database | 177 MBM-patients:  - Median (range) age: 58.0 (21-85)  - Male/female (*N*): 99/78 | - SRS: 22.8%  - WBRT: 23.7%  - Surgery ± RT: 15.8% | IPI (55.8%), nivolumab (18.9%), pembrolizumab (25.3%) | Median (95% CI) OS: 10.6 (8.7-12.5) months |  | Distant control rate:  - Overall: 34.2%  - Single MBM: 46.2%  - Multiple MBM: 30.7% |  |  |
| 64 | Murphy et al., 2018 | Retrospective review of database | 26 MBM-patients:  - Mean age: 57  - Male/female (*N*): 18/8 | Unknown | SRS combined with IPI, nivolumab and pembrolizumab | Median OS overall: 26.1 months  Median OS-rates overall:  - 1-year: 68.8%  - 2-year: 55.6%  - 3-year: 44.5% | 2-year LC: 95.4% |  | Median intracranial PFS:  - Concurrent: 19 months  - Nonconcurrent: 3.4 months  *P*<0.0001 | No grade 4 or 5 AE’s  Grade 3 CNS hemorrhages: 2/26 (7.7%) |
| 65 | Narayana et al., 2013 | Retrospective chart review | 12 MBM-patients  - Median (range) age: 48.5 (30-91) | - Systemic: 9/12 (75.0); 7/9 (77.8) IPI | RT combined with Vemurafenib | 6-month OS: 92% | 6-month LC: 75% |  | 6-month freedom from new MBM: 57% | Radiation necrosis: 1/12 (8.3%) |
| 66 | Nardin et al., 2017 | Retrospective review of database | 25 MBM-patients  - Median (range) age: 58 (32-80)  - Male/female (*N*): 14/11 | - Surgery: 4/25 (15)  - SRS: 5/25 (20)  - WBRT: 4/25 (15)  - CT: 7/25 (28)  - IPI: 15/25 (60)  - BRAFi: 6/25 (24)  - BRAF/MEKi: 5/25 (20) | SRS combined with pembrolizumab | Median (95% CI) OS after:  - Diagnosis of MBM: 15.3 (4.6-26) months  - SRS: 14.6 (5.6-23.6) months  - SRS + pembro: 11 (5.3-16.7) months  Median OS-rates:  - 6-months: 72%  - 12-months: 49% | LC in 17/25 (68%) patients after median of 8.4 months follow-up | Distant progression in 16/25 (64%) after a median of 3 months | Intracranial PFS (95% CI): 4 (2.2-5.8) months | Grade 3 radiation necrosis: 3/25 (12%) |
| 67 | Olson et al., 2016 | Retrospective review of database | 26 MBM-patients; 14/26 SRS before/during IPI, 12/26 SRS after IPI  - Median age: 63  - Male/female (*N*): 14/13 | - Systemic: 9/26 (34.6) | SRS combined with IPI | Median (95% CI) OS following initial SRS: 10.4 (CI 6.5-23.4) months  *SRS before/during IPI:*  - Median (95% CI) OS: 23.4 (5.7-NA) months  *SRS after IPI:*  - Median (95% CI) OS: 10.4 (1.9-NA) months |  |  | Median (95% CI) extracranial PFS: 3.0 (1.6-4.6) months  Median (95% CI) intracranial PFS: 6.3 (3.1-12.2) months  6-month (95% CI) distant intracranial PFS: 44% (22-64)  12-month (95% CI) distant intracranial PFS: 26% (9-47)  *SRS alone:*  - Median (95% CI) intracranial PFS: 3.3 (2.8-NA) months  - 6-month (95% CI) intracranial PFS: 34% (12-59)  *Addition of WBRT:*  - Median (95% CI) intracranial PFS: 7.5 (0.8-18.7) months  - 6-month (95% CI) intracranial PFS: 67% (19-90)  *SRS before/during IPI (N*=14*), distant intracranial progression:*  - Median (95% CI) intracranial PFS: 4.0 (1.8-18.7) months  *SRS after IPI (N*=12*), distant intracranial progression:*  - Median (95% CI) intracranial PFS: 5.2 (1.9-not reached) months | - Grade 3 CNS toxicity: 3/26 (11.5%)  - Radionecrosis: 2/26 (7.7%) |
| 68 | Ostheimer et al., 2015 | Retrospective review of database | 100 MBM-patients  - Median (range) age: 57 (27-81)  - Male/female (*N*): 65/35 | - Systemic: 50/100 (50.0) | - WBRT only: 10/100 (10.0)  - Systemic only: 9/100 (9.0)  - Local only: 16/100 (16.0)  - WBRT + systemic:19/100 (19.0)  - WBRT + local: 3/100 (3.0)  - Systemic + local: 15/100 (15.0)  - WBRT + systemic + local: 12/100 (12.0)  - No: 16/100 (16.0)  Systemic agents used:  - TMZ: 41/100 (41.0)  - Fotemustine: 18/100 (18.0)  - Both: 3/100 (3.0)  - Neither: 44/100 (44.0) | *Entire cohort:*  - Median OS: 3.9 months  - 1-year survival rate: 21.4%  *Local (either surgery and/or SRS):*  - Median OS: 6.9 months (46/100)  *No local therapy:*  - Median OS: 2.6 months (54/100)  (*P*<0.001)  *Systemic therapy:*  - Median OS: 5.1 months (55/100)  *No systemic therapy:*  - Median OS: 3.1 months (45/100)  (*P*=0.002)  *WBRT + local therapy:*  - Median OS: 5.6 months  *Systemic + local therapy:*  - Median OS: 14.2 months | Median LC:  - Surgery: 2.8 months  - Local therapy + SRS: 2.5 months  - Local therapy + surgery + SRS: 13.2 months  - Local therapy + systemic therapy: 11.1 months |  |  |  |
| 69 | Parakh et al., 2017 | Retrospective review of database | 66 MBM-patients:  - Median (range) age: 62 (19-85)  - Male/female (*N*): 45/21 | - Surgery: 3/66 (5)  - SRS: 9/66 (14)  - WBRT: 12/66 (18)  - Combination: 18/66 (27) | Nivolumab or pembrolizumab | Median (95% CI) OS: 9.9 (6.9-17.7) months  Median OS:  - Symptomatic MBM: 5.7 months  - Asymptomatic MBM: 13.0 months  *P*=0.068  - Pts on cortico’s: 4.8 months  - Pts not on cortico’s: 13.1 months  *P*=0.039  - Prior SRS or WBRT: 13.1 months  - No prior SRS or WBRT: 8.3 months  *P*=0.201  - Subsequent RT: 13.0 months  - No subsequent RT: 8.3 months  *P*=0.192 | Intracranial disease control: 37/66 (56%) | Extracranial response: 38% patients | Median (95% CI) intracranial PFS: 5.3 (3.3-8.2) months  Median intracranial PFS:  - Prior SRS or WBRT: 5.3 months  - No prior SRS or WBRT: 6.4 months  *P*=0.645  - Subsequent RT: 5.3 months  - No subsequent RT: 6.4 months  *P*=0.770 |  |
| 70 | Patel et al., 2016 | Retrospective review of database | 87 MBM-patients; 72/87 SRS, 15/87 SRS + BRAFi  *SRS:*  - >65 years (n): 24/72  - Male/female (*N*): 55/17  *SRS + BRAFi:*  - >65 years (n): 4/15  - Male/female (*N*): 11/4 | *SRS:*  - Systemic: 40/72 (55.6)  - CT: 21/72 (29.2)  *SRS + BRAFi:*  - Systemic: 11/15 (73.3)  - CT: 0/15 (0.0) | SRS combined with BRAFi | *SRS:*  - 6-month OS: 72.8%  - 12-month OS: 40.4%  *SRS + BRAFi:*  - 6-month OS: 78.6%  - 12-month OS: 64.3%  (*P*=0.20) | Median (range) time to local recurrence (LR): 4.37 months (0-18)  1-year LR rate:  - SRS: 3.3%  - SRS + BRAFi: 9.6%  (*P*=0.43) |  |  | 1-year radiation necrosis:  - SRS: 11.0%  - SRS + BRAFi: 22.2%  (*P*<0.001)  Median time to RN: 8.1 months |
| 71 | Patel et al., 2015 | Retrospective review of database | 54 MBM-patients; 34/54 SRS, 20/54 SRS + IPI  *SRS:*  - Median age: 60.2  - Male/female (*N*): 28/6  *SRS + IPI:*  - Median age: 56.5  - Male/female (*N*): 13/7 | *SRS:*  - BRAFi: 4/34 (11.8)  - Systemic: 17/34 (50.0)  *SRS + IPI:*  - BRAFi: 7/20 (35.0)  - Systemic: 15/20 (75.0) | SRS and SRS combined with IPI | 1-year OS:  - SRS: 38.5%  - SRS + IPI: 37.1%  (*P*=0.84)  *IPI within 14 days of SRS:*  - 1-year OS: 42.9%  - 2-year OS: 42.9%  *IPI > 14 days of SRS:*  - 1-year OS: 33.8%  - 2-year OS: 16.9%  *SRS alone:*  - 1-year OS: 38.5%  - 2-year OS: 25.7% | 1-year LC rate:  - SRS: 92.3%  - SRS + IPI: 71.4%  (*P*=0.40) |  |  | 1-year radiation necrosis:  - SRS: 20.92%  - SRS + IPI: 30.0%  (*P*=0.078)  1-year haemorrhage rate:  - SRS: 14.7%  - SRS + IPI: 15.0%  (*P*=1.00) |
| 72 | Qian et al., 2016 | Retrospective review of database | 75 MBM-patients  - Mean age: 62.5  - Male/female (*N*): 51/24 | - CT: 18/75 (24.0)  - BRAFi: 15/75 (20.0) | SRS combined with immunotherapy (54/75 (72%) anti-CTLA-4, 21/75 (28%) anti-PD-1) | Median (range) OS all patients: 18.5 (2.1-96.1) months  *Anti-CTLA-4:*  - Median (range) OS for non-concurrent treatment: 8.0 (2.1-61.8) months  - Median (range) OS for concurrent treatment: 19.1 (3.3-64.2) months  *Anti-CTLA-4 and anti-PD-1:*  - Median (range) OS for non-concurrent treatment: 9.0 (2.1-61.8) months  - Median (range) OS for concurrent treatment: 19.1 (2.7-64.2) months |  |  |  |  |
| 73 | Queirolo et al., 2014 | Retrospective analysis of database | 146 MBM-patients  - Median (range) age: 54 (17-78)  - Male/female (*N*): 76/70 | - Dacarbazine: 52/146 (35.6)  - Fotemustine: 59/146 (40.4)  - TMZ: 79/146 (54.1)  - Platinum-based chemo: 54/146 (37.0)  - Interferon: 20/146 (13.7)  - BRAFi: 22/146 (15.1)  - RT: 6/146 (4.1) | IPI | Median (95% CI) OS: 4.3 (3.4-5.2) months  1-year (95% CI) OS rate: 20% (13-26)  Median (range) OS with prior RT: 5.5 (2.2-6.7) months  Median OS with prior interferon: 4.1 months |  |  | Median (95% CI) PFS: 3.1 (2.7-3.5) months  1-year (95% CI) PFS rate: 17% (10-23) | Treatment related AE of any grade: 42/146 (28.8%)  Treatment-related AES grade 3/4: 9/146 (6.2%)  Liver dysfunction: 4/146 (2.7%)  Diarrhoea: 2/146 (1.4%) |
| 74 | Rahman et al., 2018 | Retrospective review of database | 74 MBM-patients  *Concurrent IT/RT (35/74)*  - Median (IQR) age: 66.7 (60.9-73.8)  *Nonconcurrent IT/RT (39/74)*  - Median (IQR) age: 59.9 (49.3-69.8) | *Concurrent IR/IT*  - Surgery: 10/35 (29)  *Nonconcurrent IR/IT*  - Surgery: 6/39 (15)  - BRAFi: 14/39 (36) | SRS/SRT + IT  *Concurrent IT/RT*  - IPI: 24/35 (69%)  - Pembrolizumab: 7/35 (20%)  - IPI+nivolumab: 2/35 (6%)  - Nivolumab: 1/35 (3%)  - Investigational anti-PD-L1: 1/35 (3%)  *Nonconcurrent IT/RT*  - IT prior to RT: 15/39 (38%)  - IT after RT: 24/39 (62%) | Median OS:  - Overall: 13.9 months  - Concurrent: 17.8 months  - Non-concurrent: 11.6 months |  |  | Median intracranial PFS: 2.9 months | Symptomatic radiation necrosis:  - Overall: 9/74 (12.2%)  - Concurrent: 4/35 (11.4%)  - Nonconcurrent: 5/39 (12.8%) |
| 75 | Robin et al., 2018 | Retrospective review of database | 38 MBM-patients  *Anti-PD1* ± anti-CTLA4  - <70 years: 11/13  - ≥70 years: 2/13  - Male/female (*N*): 8/5  *Anti-CTLA4*  - <70 years: 19/25  - ≥70 years: 6/25  - Male/female (*N*): 16/9 |  | SRS combined with anti-CTLA4 (66%) ± anti-PD1 (34%) | Median OS: not reached | 2-year LC:  - Patient-based: 81%  - Lesion-based: 92% |  | Median PFS:  - Overall: 3.4 months  - Anti-PD1 or combination Tx: 20.3 months  - Anti-CTLA4: 2.4 months  *P*=0.043 | Grade 3 radiation necrosis:  - Anti-CTLA4: 3/38 (8%)  - IPI: 0 |
| 76 | Schmidberger et al., 2018 | Retrospective review of database | 41 MBM-patients  *IPI before RT (20/41)*  - Median age: 62.5  - Male/female (*N*): 14/6  *IPI after RT (21/41)*  - Median age: 52  - Male/female (*N*): 14/7 | *IPI before RT*  - IL-2/interferon α/CT/vemurafenib: 16/20 (80)  *IPI after RT*  - IL-2/interferon α/CT/vemurafenib: 17/21 (81) | WBRT or SRS combined with IPI  - WBRT: 19/41  - SRS: 15/41  - Combination: 7/41 | Median OS:  - RT + IPI 9.0 months  - WBRT: 3.0 months  *P*=0.00003  Median OS:  - IPI after RT: 11.0 months  - IPI before RT: 3.0 months  *P*=0.015 |  |  | Median intracranial PFS: 3.0 months  Median intracranial PFS:  - IPI after RT: 6.0 months  - IPI before RT: 2.0 months  *P*=0.019 | No grade 4 AE’s |
| 77 | Seifert et al., 2015 | Retrospective review of database | 68 patients, 14/68 MBM  - Median (range) age: 53 (18-77)  - Male/female (*N*): 39/29 | - Systemic: 21/68 (30.9) | Vemurafenib | Median (95% CI) OS with progression within CNS: 6.0 (2.1-9.9) months |  |  |  |  |
| 78 | Siena et al., 2010 | Phase 2 study | 53 MBM-patients  - Mean (SD) age: 51.1 (± 11.0)  - Male/female (*N*): 34/19 | - CT: 21/53 (39.6)  - RT: 2/53 (3.8)  - WBRT: 14/53 (26.4) | Temozolomide | Median OS: 3.3 months |  |  | Median PFS: 1.9 months | All grade AEs:  - Lymphopenia: 16/53 (30.2%)  - Thrombocytopenia: 13/53 (24.5%)  - Nausea: 13/53 (24.5%)  - Vomiting: 14/53 (26.4))  - Headache: 10/53 (18.9%)  - Asthenia: 9/53 (17.0%) |
| 79 | Silk et al., 2013 | Retrospective review of database | 70 MBM-patients; 37/70 No IPI, 33/70 IPI  *No IPI:*  - Mean age: 57.7  - Male/female (*N*): 20/17  - WBRT (*N*): 21/37  -SRS (*N*): 16/37  *IPI:*  - Mean age: 56.6  - Male/female (*N*): 20/13  - WBRT (*N*): 16/33  - SRS (*N*): 17/33 | *No IPI:*  - Craniotomy: 7/37 (18.9)  - Systemic: 19/37 (51.4)  *IPI:*  - Craniotomy: 6/33 (18.2)  - Systemic: 14/33 (42.4) | RT combined with IPI | Median (95% CI) OS:  - No IPI: 5.3 (4.0-7.6) months  - IPI: 18.3 (8.1-25.5) months  *IPI before RT:*  - Median OS: 8.1 months  *IPI after RT:*  - Median OS: 18.4 months  SRS vs. WBRT: HR = 0.45  (*P*=0.008)  *IPI + WBRT:*  - Median OS: 3.1 months  *WBRT:*  - Median OS: 5.3 months  (*P*=0.60)  *IPI + SRS:*  - Median OS: 19.9 months  *SRS:*  - Median OS: 4.0 months  (*P*=0.009) | PR):  - WBRT: 5/27 (18.5%)  - SRS: 3/17 (17.6%)  - No IPI: 2/22 (9.1%)  - IPI before RT: 4/10 (40.0%)  - IPI after RT: 2/12 (16.7%) |  |  |  |
| 80 | Skrepnik et al., 2017 | Retrospective review of database | 25 MBM-patients  - Median (range) age: 68.5 (33-85)  -Male/female (*N*): 20/5 | - Surgery: 2/25 (8.0)  - BRAFi: 3/25 (12.0)  - Interleukin/  Interferon: 4/25 (16.0)  - Pembrolizumab: 6/25 (24.0) | SRS combined with IPI | Median OS: 35.8 months  1-year OS: 83%  2-year OS: 64% | LC: 55/58 lesions (94.8%) after a median follow-up of 22.7 months |  |  | Radiation necrosis: 12/58 lesions (20.7) |
| 81 | Sloot et al., 2018 | Retrospective cohort study | 243 MBM-patients  - Median (range) age: 58 (15-86)  - Male/female (*N*): 159/84 | - Craniotomy: 37/243 (15.2)  - WBRT: 38/243 (15.6)  - SRS: 118/243 (48.6) | - BRAFi: 39/243 (16.0)  - IL-2: 35/243 (14.4)  - Anti-CTLA4: 77/243 (31.7)  - Anti-PD1: 20/243 (8.2) | Median (95% CI) OS from:  - First MBM: 10.5 (8.6-12.8) months  - First systemic Tx: 14.7 (13.0-21.5) months  Median OS from:  - 2000-2008: 7.5 months  - 2009-2010: 8.5 months  - 2011+: 22.7 months  *P*=0.002  OS rates (95% CI):  - 1-year: 43.4% (36.6-50.1%)  - 2-year: 27.3% (20.5-34.4)  - 3-year: 17.5% (11.3-24.9%) |  |  |  |  |
| 82 | Staudt et al., 2010 | Retrospective review of database | 265 MBM-patients  - Median age: 58  - Male female (*N*): 154/111 | - Systemic (CT or interferon): 128/265 (48.3) | - Surgery: 63/265 (23.8)  - SRS: 31/265 (11.7)  - WBRT: 122/265 (46.0)  - Chemo: 28/265 (10.6)  - No: 12/265 (4.5) | Median (95% CI) OS entire cohort: 5.0 (4.3-5.7) months  Median (95% CI) OS:  - Surgery: 9.0 (7.0-11.0) months  - SRS: 9.0 (7.0-11.0) months  - WBRT: 4.0 (3.2-4.8) months  - Chemo: 3.0 (1.6-4.4) months  - No: 1.0 (0.1-1.9) months |  |  |  |  |
| 83 | Stokes et al., 2017 | Retrospective review of database | 1287 MBM-patients  *No IT (1102/1287):*  - <60 years: 472/1102  - 60-69 years: 313/1102  - ≥ 70 years: 317/1102  - Male/female (*N*): 800/302  *IT (185/1287):*  - <60 years: 86/185  - 60-69 years: 55/185  - ≥ 70 years: 44/185  - Male/female (*N*): 143/51 | CT:  - No IT: 495/1102 (44.9)  - IT: 28/185 (15.1) | RT combined with IT  RT:  - Conventionally fractioned: 818/1287  - SRS: 469/1287 | Median (95% CI) OS:  - No IT: 6.1 (5.6-6.6) months  - IT: 10.8 (9.1-12.5) months  *P*<0.01 |  |  |  |  |
| 84 | Szyszka-Charewicz, 2016 | Retrospective review of database | 110 MBM-patients  - Mean (range) age: 55 (34-72)  - Male/female (*N*): 66/44 | Unknown | WBRT combined with:  - Chemo (TMZ): 14/110 (12.7)  - Surgery: 12/110 (10.9)  - SRS: 8/110 (7.3) | Median (95% CI) OS***: 4.8 (4.1-5.2) months  Median OS:  - WBRT alone: 4.0 months  - WBRT + surgery: 9.2 months  - WBRT + SRS: 7.4 months  - WBRT + TMZ: 3.8 months  6-month survival: 39.1%  12-month survival: 13.6% |  |  |  |  |
| 85 | Tawbi et al., 2018 | Phase 2 study | 94 MBM-patients  - Median (range) age: 59 (22-81)  - Male/female (*N*): 65/29 | - SRS: 8/94 (9)  - Systemic Tx: 16/94 (17) | IPI combined with nivolumab | Median OS-rate:  - 6-month: 92.3%  - 9-month: 82.8%  - 12-month (estimated): 81.5% | Intracranial response (95% CI): 55% (45-66%) |  | Intracranial PFS rate:  - 6-month: 64.2%  - 9-month: 59.5%  Extracranial PFS rate:  - 6-month: 75.9%  - 9-month: 70.4%  Global PFS rate:  - 6-month: 61.1%  - 9-month: 56.6% | Grade 3/4 AE’s:  - Overall: 52/94 (55%)  - Headache: 3/94 (3%)  - Brain oedema: 2/94 (2%)  - Intracranial haemorrhage: 1/94 (1%)  - Peripheral motor neuropathy: 1/94 (1%)  - Syncope: 1/94 (1%)  Grade 5 immune-related myocarditis leading to death: 1/94 (1%) |
| 86 | Tazi et al., 2015 | Retrospective review of database | 31 patients;  10/31 MBM, 21/31 no MBM  *MBM:*  - Median (range) age: 65.5 (41-81)  - Male/female (*N*): 5/5  *No MBM:*  - Median (range) age: 64.0 (43-89)  - Male/female (*N*): 16/5 | Unknown | SRS combined with IPI | *MBM***:*  - Median OS: 29.3 months  - 3-year (95% CI) survival rate from cycle 1 IPI: 50% (27-93)  *No MBM***:*  - Median OS: 33.1 months  - 3-year (95% CI) survival rate from cycle 1 IPI: 39% (19-81) |  |  |  | *MBM:*  - Grade 3 diarrhea: 1/10 (10.0%)  *No MBM:*  - Grade 3 diarrhea: 3/21 (14.3%)  - Grade 3 hypopituitarism: 1/21 (4.8%) |
| 87 | Tio et al., 2017 | Retrospective review of database | 355 MBM-patients  - Median age: 66  - Male/female (*N*): 251/104 | Unknown | Surgery (42%), SRS (21%), WBRT (34%), CT (10%), BRAFi±MEK (26%), IPI (15%), anti-PD1 (4%), IPI+anti-PD1 (2%) | Median (95% CI) OS:  - BSC: 1.8 (1.2-2.3) months  - Systemic Tx: 5.4 (3.1-7.7) months  - WBRT: 4.4 (2.4-6.3) months  - Systemic Tx + WBRT: 5.2 (4.1-6.4) months  - SRS and/or surgery ± WBRT: 6.4 (5.4-7.5) months  - Systemic Tx + SRS and/or surgery: 14.9 (10.7-19.0) months |  |  |  |  |
| 88 | Trommer-Nestler et al, 2018 | Retrospective review of database | 26 MBM-patients  *SRS (13/26):*  - Mean age: 54.9  - Male/female (*N*): 6/7  *SRS + anti-PD1 (13/26):*  - Mean age: 61.5  - Male/female (*N*): 8/5 | *SRS*  - BRAFi: 8/13 (62)  - MEKi: 2/13 (15)  - TK/RTKi: 2/13 (15)  - CT: 6/13 (46)  - Interferon: 0/13 (0)  - IPI: 4/13 (31)  *SRS + anti-PD1*  - BRAFi: 3/13 (23)  - MEKi: 3/13 (23)  - TK/RTKi: 0/13 (0)  - CT: 4/13 (31)  - Interferon: 2/13 (15)  - IPI: 6/13 (46) | SRS combined with anti-PD1 (pembrolizumab 10/13, nivolumab 3/13) |  | 6-month LC:  - SRS: 80%  - SRS + anti-PD1: 86% |  |  |  |
| 89 | Vecchio et al., 2013 | Retrospective review of database | 115 MBM-patients  - ≥ 53 years (n): 59/115  - Male/female (*N*): 68/47 | - Surgery: all except patients with unknown primary tumor  - IT: 34/115 (29.6)  - CT: 11/115 (9.6)  - Immunochemo: 31/115 (27.0)  - No: 39/115 (33.9) | - Surgery or SRS (± CT or RT): 28/115 (24.3)  - Chemo ± RT: 48/115 (41.7)  - RT ± support: 9/115 (7.8)  - Support: 29/115 (25.2) | Median (95% CI) OS:  - Overall: 4.3 (2.6-6.1) months  - Surgery or SRS (± CT or RT): 10.8 (3.8-17.9) months  - Chemo ± RT: 5.4 (3.3-7.5) months  - RT ± support: 2.5 (2.1-2.9) months  - Support: 1.3 (0.8-1.8) months |  |  |  |  |
| 90 | Vosoughi et al., 2018 | Retrospective review of database | 79 MBM-patients  - Median (range) age: 63 (17-91)  - Male/female (*N*): 53/26 | - Systemic Tx: 49/79 (62.0) | - Craniotomy: 34/79 (43.0)  - WBRT: 16/79 (20.3)  - SRS: 54/79 (68.4)  - IPI: 39/79 (49.4)  - Anti-PD1: 28/79 (35.4)  - BRAF±MEKi: 24/79 (30.4)  - CT: 35/79 (44.3)  - IL-2: 10/79 (12.7)  *Combinations of treatment unknown | Median (range) OS from:  - Initial MBM: 12.8 (1.1-71.9) months  - Initial melanoma: 60.5 (5.5-367.1) months  - First craniotomy: 17.3 (2.4-60.7) months  - First SRS: 15.4 (1.2-71.8) months  - First WBRT: 6.8 (2.2-12.5) months  Median (range) OS:  - anti-CTLA4 before MBM: 10.5 (2.0-55.3) months  - anti-CTLA4 after MBM: 19.2 (1.2-65.0) months  - Anti-PD1 before: 8.5 months  - Anti-PD1 after MBM: 37.9 (5.3-65.0) months  - BRAF±MEKi before MBM: 10.9 (2.1-55.3) months  - BRAF±MEKi after MBM: 12.7 (2.7-70.9) months |  |  |  |  |
| 91 | Wattson et al., 2015 | Retrospective review of database | 106 MBM-patients;  37/106 MAPKi, 69/106 no MAPKi  *MAPKi:*  - Median age: 56.7  - Male/female (*N*): 21/16  *No MAPKi:*  - Median age: 60.0  - Male/female (*N*): 47/22 | SRS and/or resection:  - MAPKi: 21/37 (56.8)  - No MAPKi: 43/69 (62.3)  WBRT:  - MAPKi: 16/37 (43.2)  - No MAPKi: 33/69 (47.8)  IT  - MAPKi: 20/37 (54.1)  - No MAPKi: 65/69 (94.2)  CT:  - MAPKi: 12/37 (32.4)  - No MAPKi: 27/69 (39.1) | MAPKi:  - Vemurafenib: 24/37 (64.9)  - Dabrafenib + Trametinib: 13/37 (35.1) | Median OS***:  - MAPKi: 14.1 months  - No MAPKi: 7.0 months (*P*=0.03)  Median OS MAPKi:  - Tx after BM: 16.6 months  - Tx prior to BM: 5.6 months (*P*=0.03)  1-year (95% CI) survival:  - MAPKi: 59% (41-73)  - No MAPKi: 27% (17-38) |  |  | PFS (95% CI):  *MAPKi:*  - Tx after BM: 7.1 (0.8-3.9) months  - Tx prior to BM: 2.1 (0.8-3.9) months |  |
| 92 | Williams et al., 2017 | Phase 1 study | 16 MBM-patients  *SRS + IPI (11/16):*  - Median (SD) age: 57 ± 13  - Male/female (*N*): 8/3  *WBRT + IPI (5/16):*  - Median (SD) age: 63 ± 5  - Male/female (*N*): 4/1 | Surgery:  - SRS+IPI: 6/11 (55)  - WBRT+IPI: 3/5 (60) | WBRT/SRS combined with IPI | Median OS:  - SRS+IPI: not reached  - WBRT+IPI: 8 months |  |  | Median PFS:  - SRS+IPI: 2.1 months  - WBRT+IPI: 2.5 months | Grade 3 AE’s SRS+IPI:  - Headache: 1  - Diarrhea: 4  - Anemia: 1  Pleuritic pain: 1  Grade 3 AE’s WBRT+IPI:  - Fatigue: 1  - Diarrhea: 1  - Lymphopenia: 1  - Hypophysitis: 1 |
| 93 | Wilkins et al., 2015 | Retrospective review of database | 393 MBM-patients;  231/393 cohort 1, 162/393 cohort 2  *Cohort 1:*  - Median (range) age: 55 (17-92)  - Male/female (*N*): 153/78  *Cohort 2:*  - Median (range) age: 58 (22-89)  - Male/female (*N*): 70/92 | Unknown | *Cohort 1: RT ± surgery and drugs*  - WBRT: 226/231 (97.8)  - SRS: 24/231 (9.1)  - Surgery: 39/231 (16.9)  - Chemo: 67/231 (29.0)  *Cohort 2: targeted therapy and best supportive care*  - WBRT: 104/162 (64.2)  - SRS: 27/162 (16.7)  - Surgery: 26/162 (16.0)  - Chemo: 31/162 (19.1)  - Targeted: 41/162 (25.3)  - BSC: 20/162 (12.3) | Median (95% CI) OS:  - Cohort 1: 3.6 (3.5-4.8) months  - Cohort 2: 4.4 (3.5-4.8) months |  |  |  |  |
| 94 | Wolf et al., 2016 | Prospective review of database | 80 MBM-patients; 35/80 BRAF-mutation, 45/80 no BRAF-mutation  *BRAF-mutation:*  - Mean (SD) age: 52.5 (± 14.0)  - Male/female (*N*): 16/19  *No BRAF-mutation:*  - Mean (SD) age: 65.5 (± 12.5)  - Male/female (*N*): 34/11 | WBRT:  - BRAF-mutation: 3/35 (8.6)  - No BRAF-mutation: 4/45 (8.9)  Surgery:  - BRAF-mutation: 6/35 (17.1)  - No BRAF-mutation: 13/45 (28.9) | SRS combined with BRAFi (if BRAF-mutation) | Median (95% CI) OS entire cohort*: 6.7 (5.3-8.1) months  Median (95% CI) OS entire cohort***: 9.7 (6.7-12.6) months  Median (95% CI) OS BRAF-mutation*: 11.2 (5.3-17.0) months  Median (95% CI) OS BRAF-mutation***: 13.2 (8.3-18.1) months  Median (95% CI) OS no BRAF-mutation*: 4.5 (2.5-6.5) months  Median (95% CI) OS no BRAF-mutation***: 6.9 (4.4-9.3) months  *BRAF-mutation:*  - 6-month survival rate: 54%  - 12-month survival rate: 41%  *No BRAF-mutation:*  - 6-month survival rate: 28%  - 12-month survival rate: 19% | Total (SD) LC-rate: 92.5% (± 22.3) |  | Overall (range) median time to intracranial progression: 2.2 (0.4-16.6) months  Time to progression (range):  - BRAF-mutation: 3.9 (0.8-16.6) months  - No BRAF-mutation: 1.7 (0.4-9.3) months  (*P*=0.02) | Intratumoral haemorrhage rate per patient: 9/67 (13.8%) |
| 95 | Xu et al., 2017 | Retrospective review of database | 65 MBM-patients;  13/65 Group A (BRAF-mutation, no BRAFi), 17/65 Group B (BRAF-mutation and BRAFi), 35/65 Group C (wild-type BRAF)  *Group A:*  - Median (range) age: 58 (20-80) - Male/female (*N*): 9/4  *Group B:*  - Median (range) age: 56 (24-73)  - Male/female (*N*): 11/6  *Group C:*  - Median (range) age: 60 (31-81)  - Male/female (*N*): 27/8 | - WBRT: 10/65 (15.4)  - Surgery: 5/65 (7.7) | SRS combined with BRAFi (12/17 Vemurafenib, 5/17 Dabrafenib) | Median (range) OS entire cohort***: 9 months (7.3-10.7)  *Group A***:*  - Median (range) OS: 3.0 (0-6.5) month  - 1-year survival: 39%  - 2-year survival: 19%  *Group B***:*  - Median (range) OS: 23.0 (0-46.4) months  - 1-year survival: 58%  - 2-year survival: 44%  *Group C***:*  - Median (range) OS: 8.0 (6.2-9.8) months  - 1-year survival: 30%  - 2-year survival: 0.04% | LC for SRS entire cohort: 89.4%  1-year LC:  - Group A: 82.4%  - Group B: 92%  - Group C: 69.2% |  |  | Intratumoral haemorrhage:  - Group A: 1/13 (7.7%)  - Group B: 5/17 (29.4%)  - Group C: 3/35 (8.6%) |
| 96 | Yusuf et al., 2017 | Prospective review of database | 51 MBM-patients  - Median (range) age: 63.6 (35.2-82.5) | - WBRT: 3/51 (5.9) | SRS or SRS combined with immune checkpoint therapy (ICT):  - Peri-SRS ICT: 18/51 (35.3)  More specific:  - ICT before or during SRS: 7/51 (13.7)  - ICT after SRS: 11/51 (21.6) | Median (range) OS entire cohort: 7.1 (0.9-51.8) months  *Peri-SRS ICT:*  - Median (range) OS: 7.4 (0.9-26.4) months  *SRS alone:*  - Median (range) OS: 7.1 (1-51.8) months | Local failure entire cohort:  - 6-month LF: 21.9%  - 12-month LF: 25.2% | Freedom from distant brain failure entire cohort:  - 6-months: 34.5%  - 12-months: 15.6% |  | Radiation necrosis:  - 8.4% of the lesion sites |

Supplemental 3. Study characteristics

*****From the date of SRS ** mWHO criteria *** From the date of diagnosis MBM or stage IV

**Abbreviations:**

| MBM: Melanoma brain metastases | CT: Chemotherapy | IPI: Ipilimumab | LR: Local recurrence | PFS: Progression-free survival |
| --- | --- | --- | --- | --- |
| IT: Immunotherapy | SRS: Stereotactic radiosurgery | OS: Overall survival | LF: Local failure | AE: Adverse event |
| TT: Targeted therapy | WBRT: Whole brain radiotherapy | LC: Local control | DC: Distant control |  |
